# The E^rns^ Carboxyterminus: Much More Than a Membrane Anchor

**DOI:** 10.3390/v13071203

**Published:** 2021-06-23

**Authors:** Birke Andrea Tews, Anne Klingebeil, Juliane Kühn, Kati Franzke, Till Rümenapf, Gregor Meyers

**Affiliations:** 1 Institut für Infektionsmedizin, Friedrich-Loeffler-Institut, D-17493 Greifswald, Germany; birke.tews@fli.de (B.A.T.); kati.franzke@fli.de (K.F.); 2 Institut für Immunologie, Friedrich-Loeffler-Institut, D-17493 Greifswald, Germany; a.klingebeil@googlemail.com (A.K.); kuehnjuliane2609@gmail.com (J.K.); 3 Department of Pathobiology, Institute of Virology, University of Veterinary Medicine Vienna, 1210 Vienna, Austria; Till.Ruemenapf@vetmeduni.ac.at

**Keywords:** pestivirus, RNA virus polyprotein processing, membrane anchor, charge zipper, amphipathic helix, signal peptidase, secreted T2 RNase, ER retention, extracellular vesicles, virus assembly, virus budding, transport of virus particles

## Abstract

Pestiviruses express the unique essential envelope protein E^rns^, which exhibits RNase activity, is attached to membranes by a long amphipathic helix, and is partially secreted from infected cells. The RNase activity of E^rns^ is directly connected with pestivirus virulence. Formation of homodimers and secretion of the protein are hypothesized to be important for its role as a virulence factor, which impairs the host’s innate immune response to pestivirus infection. The unusual membrane anchor of E^rns^ raises questions with regard to proteolytic processing of the viral polyprotein at the E^rns^ carboxy-terminus. Moreover, the membrane anchor is crucial for establishing the critical equilibrium between retention and secretion and ensures intracellular accumulation of the protein at the site of virus budding so that it is available to serve both as structural component of the virion and factor controlling host immune reactions. In the present manuscript, we summarize published as well as new data on the molecular features of E^rns^ including aspects of its interplay with the other two envelope proteins with a special focus on the biochemistry of the E^rns^ membrane anchor.

## 1. Introduction

Pestiviruses are unique in the family Flaviviridae because they express three structural glycoproteins, E^rns^, E1 and E2, that all are essential for generation of infectious viruses [1,2]. Comparison of the features and known functions of these proteins with those from the related hepaciviruses reveals that pestiviral E1 and E2 are basically equivalent to the hepaciviral proteins with the same names whereas E^rns^ obviously represents a unique product. This conclusion is supported by data showing that in pseudotyped viruses pestiviral E1 and E2 are sufficient for infection of cells [3,4] whereas E^rns^ has to play some unknown additional role as a structural protein in the pestivirus life cycle. The character of E^rns^ as a unique factor becomes even more obvious when the additional features of the protein are taken into account. E^rns^ represents a virulence factor of pestiviruses [5,6,7,8]. This function depends on a highly unusual enzymatic activity, which has so far not been found in any other viral surface protein. Both sequence and structure analyses demonstrated that E^rns^ belongs to a group of evolutionary ancient RNases denoted T2 RNases [9,10,11]. The RNase activity of the protein was demonstrated with different assay systems using the purified protein, but also crude extracts of cells expressing E^rns^ [5,6,10,11,12]. As described for a number of other T2 RNases, E^rns^ is secreted from cells that produce it [13,14,15]. Secretion is only partial so that sufficient amounts of E^rns^ remain within the cell to allow efficient assembly and budding of virus particles. It is still not known in detail, which molecular principle underlies the equilibrium of retention and secretion. However, it is obvious that the highly unusual membrane anchor of the protein consisting of a long amphipathic helix that binds in plane to membrane surfaces plays a crucial role here [14,16,17]. This structure is also a key determinant for the processing of the pestivirus glycoprotein precursor since it creates a highly unusual signal peptidase processing site which is responsible for a delayed cleavage of the E^rns^-E1 precursor, which together with the requirement of E1-E2 cleavage prior to processing of E^rns^-E1 results in an ordered fashion of maturation of the envelope proteins [18,19]. 

E^rns^ is an endoribonuclease that cleaves single stranded RNA upstream of U residues but can also destroy double stranded RNA, though with reduced activity [12,20,21]. E^rns^ forms homodimers that are found within cells and virus particles but also secreted into the supernatant [7,12,20,21,22]. E^rns^ homodimer formation is crucial for the virulence of pestiviruses even though also the monomeric protein has RNase activity [7]. Many other details concerning the virulence factor activity of E^rns^ are still unclear, but the widely accepted hypothesis proposes that the secreted E^rns^ RNase is the important component. Whether E^rns^ acts in the cell free environment of the host organism or upon internalization into cells is still a matter of debate and investigation. Likewise, also the nature of the RNA that is destroyed by E^rns^ is not clear. It could be viral RNA that was taken up into a non-appropriate cellular compartment or cellular RNA in order to impair defense functions of immune cells maybe by induction of cell death. A proven result of E^rns^ RNase presence in the natural host animals is the impairment of the virus-induced type I/III interferon response. It was shown for bovine viral diarrhea virus that this repression of the interferon system represents a prerequisite for establishment of long lasting infections [23,24]. Detailed knowledge of the biochemistry of E^rns^ is a prerequisite for further elucidation of its diverse features that are important for both formation of infectious pestivirus particles and interference with the innate immune response of the host. In the present article, we summarize the data published on the biochemical features of E^rns^ and include recent results that were not published before.

## 2. Material and Methods 

### 2.1. Cells and Viruses

BHK-21 cells (kindly provided by T. Rümenapf) and chicken DF-1 cells (Collection of Cell Lines in Veterinary Medicine (CCCLV) of the Friedrich-Loeffler-Institute, Greifswald, Germany) and SK6 cells (CCCLV) were grown in Dulbecco’s modified Eagle’s medium supplemented with 10% fetal calf serum and nonessential amino acids. Two variants of modified vaccinia virus strain Ankara containing the phage T7 RNA polymerase (MVA-T7), kindly provided by G. Sutter (Ludwig-Maximilians-Universität, München, Germany) and B. Moss (National Institutes of Health, Bethesda, MD, USA) were used in our experiments [25,26]. SK6TO_E^rns^ cells were established by Co-author T. Rümenapf essentially as described before [20,27,28]. For stimulation of Erns expression doxycycline dissolved in DMSO was added to the medium to final concentration of 10 µM. The -Dox control was supplied with the equivalent volume of pure DMSO. 

### 2.2. Construction of Recombinant Plasmids

Restriction and subcloning were done according to standard procedures [29]. Unless stated otherwise, all restriction and modifying enzymes were purchased from New England Biolabs (Frankfurt, Germany) and Thermo-Fisher (Karlsruhe, Germany). Synthetic DNA oligonucleotides were synthesized by Metabion (München, Germany).

The plasmids SE^rns^ and SE^rns^-E1 used in expression studies (originally named SSeqE^rns^ and SSeqE^rns^-E1 containing the E^rns^ and E^rns^E1 coding sequences of CSFV Alfort/Tübingen [18,19]) were used as basis for the mutant expression constructs. Single and multiple point mutations were introduced with standard PCR based methods with thermostable Pfu polymerase (Promega, Heidelberg, Germany) and synthetic primers (QuikChange mutagenesis) in one single reaction or consecutive approaches. The mutated PCR products were all verified by nucleotide sequencing with the BigDye Terminator Cycle Sequencing Kit (PE Applied Biosystems, Weiterstadt, Germany). Sequence analysis and alignments were done with Geneious Prime software (Geneious Prime 2019.2.3) (Biomatters, Ltd. Auckland, New Zealand). 

For establishment of the GFP-E^rns^ expression plasmids the E^rns^ signal sequence, the E^rns^ sequence coding for the mature protein and GFP were amplified with specific primers generating overlapping sequence at the ends of the PCR products. E^rns^ was amplified from a wt expression construct and from the E^rns^ variant with a transmembrane sequence used in previous work [14]. Overlapping fragments were fused using a fusion PCR protocol and the resulting fragments were cloned into the pCI vector (Promega, Walldorf, Germany). The resulting expression plasmids contain the native E^rns^ signal sequence followed by the first three codons of the E^rns^ protein, then the GFP coding sequence and the respective E^rns^ sequences after a short linker. For the carboxy-terminal deletions the BVDV CP7 E^rns^ sequence was amplified from the full length constructs using primers that introduced stop codons into the sequences at the indicated positions. The resulting PCR products were cloned into pCITE (AGS, Heidelberg, Germany). Further details of the cloning procedures and the sequences of the primers used for cloning and mutagenesis as well as sequences and maps of the constructs are available from the authors on request.

### 2.3. Transient Expression, Immunoprecipitation and Quantification of Proteins

BHK-21 cells were infected with vaccinia virus MVA-T7, subsequently transfected with the desired cDNA construct using SuperFect (Qiagen, Hilden, Germany) and labeled with Tran35S-Label (ICN-MP Biochemicals, Eschwege, Germany or Hartmann Analytic, Göttingen, Germany) as described earlier [7]. Supernatant of the cell cultures was harvested for determination of secreted proteins, the cells were washed twice with PBS before cell extracts were prepared under denaturing conditions. Protein expression in equivalent amounts of cell-free supernatant and cell extract was analyzed via immunoprecipitation as described before [14] using monoclonal antibody 24/16 [30] for detection of E^rns^ and the E^rns^-E1 precursor. When indicated in the text precipitates were treated before electrophoresis with 1 µL PNGase F (New England Biolabs) for 1 h at 37 °C as suggested by the supplier and subsequently separated by 10% SDS-PAGE (gel system as published in [31]) and E^rns^ or E^rns^-E1 quantified with a Fujifilm BAS-1500 or a CR-35 Bio image plate scanner. The intensities of the signals were determined with TINA 2.0 or AIDA Image Analyser 5 software (equipment and software from Elysia-Raytest, Straubenhardt, Germany). For E^rns^-E1 processing studies, the E^rns^ signal was multiplied with 1.462 to correct for the lower numbers of labelled residues in E^rns^ compared to E^rns^-E1, and the corrected E^rns^ values added to those determined for uncleaved E^rns^-E1 to obtain 100% expression product as a basis for calculation the percentage of precursor. Similarly, the secretion level of E^rns^ expressed from the SE^rns^ constructs was calculated by combining the signals determined for E^rns^ in supernatant and cell extract to obtain 100% expression product for calculation of the percentage of secreted protein. The presented data represent the averages of at least three independent experiments. Statistical analysis in form of a two-tailed T test was done using the GraphPad Prism software (Statcon GmbH, Witzenhausen, Germany). The same procedure was also used for transient expression of proteins in DF-1 chicken cells with the exception that LipofectamineTM 2000 was used for transfection (protocol as recommended by supplier Invitrogen, Karlsruhe, Germany).

### 2.4. Analysis of Membrane Association 

Membrane association was essentially measured as in [7,14]. Briefly, BHK-21 cells were infected with Vaccinia MVA-T7 and transfected with expression plasmids for the different proteins. Proteins were metabolically labeled with 35S-Methionine and -Cysteine. After 24 h cell medium was collected to detect secreted proteins (Fraction 1) and transfected cells were fractionated, by shearing them first, followed by low (700× *g*, 3 min; Fraction 2) and high speed (125,000× *g*, 25 min; Fraction 3) centrifugation to pellet membranes. Soluble proteins were collected from the supernatant of the last centrifugation step (Fraction 4). Pellets were solubilized in RIP buffer and E^rns^ was precipitated from volumes corresponding to equal parts of the original samples from all fractions using 5 µL of a rabbit-anti E^rns^ serum. Precipiates were separated by SDS-PAGE. Protein amount was quantified through phosphoimaging (see description provided above) of the specific E^rns^ band in each fraction. The sum of the signal intensity in all four fractions of a sample was set to 100% and the amount that could be found in the membrane fractions (Fractions 2 and 3) was calculated accordingly. Data of at least three independent experiments is shown. Statistical analysis was done with GraphPad Prism software version 9.0.0.121 (GraphPad Software, San Diego, CA, USA) using one-way ANOVA and a Dunnett post-hoc test. 

### 2.5. Fluorescence Recovery after Photobleaching (FRAP) Experiments

Cells were seeded in eight-well coverslips (Ibidi, Gräfelfing, Germany) and transfected with expression plasmids for GFP-E^rns^ variants. Cells were imaged the next day at a Leica SP5 confocal microscope (Leica Microsystems, Wetzlar, Germany), with continuous image acquisition every ~1.3 s. GFP-Signal was bleached in specified regions with a strong light pulse and cells imaged for up to 5 min afterwards to look at signal recovery. Images were analyzed using ImageJ (ImageJ 1.53 h, National Institutes of Health, Bethesta, MD, USA)to measure fluorescence intensity per area in each image of every time series. Experiments were done in BHK-21 and U-2-OS cells (ATCC® HTB-96™, LGC Standards GmbH - Germany Office, Wesel, Germany) in at least three independent experiments and in different cells for every experiment. 

### 2.6. Recovery and Analysis of Mutant Viruses from Cloned Sequences

Mutations leading to exchanges of charged amino acids in the E^rns^ membrane anchor were introduced into the CSFV Alfort/Tübingen full-length infectious clone pA/CSFV [32] with standard procedures as described before [7]. In vitro transcription of RNA from the engineered plasmids and electroporation of cells were done as described before [7]. Replication of RNA and protein expression thereof was detected via immunofluorescence with mab A18 [33] against E2 and FITC-conjugated goat anti-mouse serum (Dianova, Hamburg, Germany). Freeze/thaw extracts were prepared from positive cultures and used for infection of fresh cells. Infection of these cells was detected via immunofluorescence as described above. RNA was isolated from the infected cells with Trizol^®^ as recommended by the supplier (Invitrogen) and subjected to RT-PCR with primers Ol-E05S (5’-CATGCCATGGGGGCCCTGTTGGCTTGGGCGGTG-3’) and Ol-HPS28.1R 5’-GGACTAGCTTTATAACCTGTCC-3’) using the OneStep RT-PCR kit (Qiagen, Hilden, Germany) and analyzed by nucleotide sequencing as described before [7]. Sequence analysis was done with the Geneious Prime software.

### 2.7. Isolation and Characterization of Extracellular Vesicles

For isolation of extracellular vesicles, the ExoQuick-TC PLUS Exosome Isolation Kit (SBI System Biosciences, Palo Alto, CA, USA) was used according to the protocol provided by the supplier. Virus particles were enriched by ultracentrifugation (SW40, 37,000 rpm, 2 h at 4 °C). The pellet fractions containing extracellular vesicles (EVs) were resuspended in PBS and further analyzed by Western blot or electron microscopy. 

For Western blot, the samples (exosome pellet, exosome preparation supernatant, cell culture supernatant or cells expressing the desired E^rns^ protein) were lysed with reducing 1×SDS sample buffer (120mM Tris-HCl, pH 6.8, 20% Glycerol, 4% SDS, 0.02% Bromphenol Blue) containing 5% β-mercaptoethanol. Separation of the proteins was done with the same gel system as described above. After the run, gels were equilibrated in transfer buffer (0.25 M Tris, 1.925 M Glycin, 0.1% SDS, 18% ethanol, pH 8.3) before blotting onto nitrocellulose membranes (GE Healthcare/Fisher Scientific, Schwerte, Germany) at 100V for 1 h in the same buffer. Membranes were blocked with 5% non-fat milk in PBS-T (PBS 0.05% Tween 20), washed with PBS-T, incubated overnight at 4 °C with rabbit antiserum K81 (raised against a peptide corresponding to the carboxy-terminus of E^rns^), or rabbit serum anti TSG101 (Sigma Aldrich, Darmstadt, Germany) at a dilution of 1:1000. Secondary antibodies α-rabbit-PO diluted 1:10 000 in PBS-T were used for detection of bound antibodies and washed with PBST. Staining of the blot was done with SuperSignal West Pico (Pierce, Rockford, IL) as recommended.

For transmission electron microscopy (TEM) the samples containing vesicles were transferred to formvar coated TEM grids (400 mesh, Plano GmbH, Wetzlar, Germany) and stained with 2% phosphotungstic acid at pH 6. 

For immunogold labelling the samples of enriched viral particles or vesicles were also transferred to formvar coated TEM grids, washed with PBS and blocked with BSA in PBS. Then the grids were incubated with the mAb 24/16 (undiluted cell culture supernatant) for 45 min and an anti-mouse immunogold conjugate (GMHL10, Plano, 1:50, Plano Wetzlar, Germany) for 45 min at room temperature. Finally, the grids were stained with 2% phosphotungstic acid at pH 7.4 for 7 min at room temperature and analyzed with a Tecnai-Spirit (FEI, Eindhoven, The Netherlands) at an accelerating voltage of 80 kV.

### 2.8. Quantification of Viral Intra- and Extracellular RNA with qRT-PCR

To analyze whether mutant RNAs not able to generate infectious viruses nevertheless secreted non-infectious viral particles containing packaged RNA we generated infectious viruses via complementation in trans. Electroporation was used to transfect the mutant RNAs into SK6TO_E^rns^ cells expressing E^rns^ under control of a doxycycline dependent promotor. The supernatant of these cells containing the complemented infectious viruses was harvested at 24 to 48h, titrated and used for infection of wt SK6 cells at an MOI of 1 for 1 h at 37 °C and then seeded in several wells. To test whether wt cells infected with the complemented mutants secreted packaged viral RNA supernatant and cells were harvested and subjected to RNA isolation 4h after infection. A second set of samples was treated equivalently but at 48h pi. RNA was isolated with QIAamp Viral RNA Mini Kit (Qiagen, Hilden, Germany) according to the manufacturer’s instruction. In short, we mixed 560 µL AVL (with 5.6 µg Carrier-RNA) buffer with cells or supernatant for the lysis of each sample. At this step, we added internal control RNA (IC2) to monitor the effective RNA-isolation. After 10 min of incubation time ethanol was added to the sample and mixed. Then the sample was applied to the QIAamp Mini columns and centrifuged for 1 min at 6000 g. The RNA sample on the column was washed with two different buffers (AW1 and AW2) and finally the RNA was eluted with AVE buffer. RNA was stored at −20 °C and the RNA amount was determined using the NanoDrop 2000 (Thermo-Fisher). 

Quantitative detection of viral RNA via real time RT-PCR with CSFV specific primers (CSF-100-F (5’-ATG CCC AYA GTA GGA CTA GCA-3’); CSF-192-R (5’-CTA CTG ACG ACT GTC CTG TAC-3’) and probe (CSF-Probe-1 (5’-*FAM*-TGG CGA GCT CCC TGG GTG GTC TAA GT-*TAMRA*-3’)) according to Hoffmann et al. [34] served as read out. The reaction was carried out using the QuantiTectTM Probe RT-PCR Kit (Qiagen). A total of 20 µL Mastermix (12.5 µL of 2× QuantiTect Probe RT-PCR Master Mix; 0.25 µL of QuantiTect RT Mix; 3.25 µL of RNase-free water; 2 µL of CSF primer mix; 2 µL of EGFP primer mix) was prepared for each sample and 5 µL of sample RNA were added. As an independent control for RNA-isolation and reliability of the PCR run internal control RNA (IC2) was added to every sample [35]. In addition to the samples, a non-template control and a positive control were run. To quantify the amount of viral RNA in the sample a standard curve was generated.

The PCR program used was as follows: reverse transcription for 30 min at 50 °C followed by activation of Taq polymerase for 15 min at 95 °C and 42 cycles of PCR with denaturation of 15 sec at 95 °C, annealing for 30 sec at 57 °C and elongation for 30 sec at 68 °C. Fluorescence data was collected in the annealing phase.

## 3. Results

### 3.1. The E^rns^ Membrane Anchor

**Figure 1:**
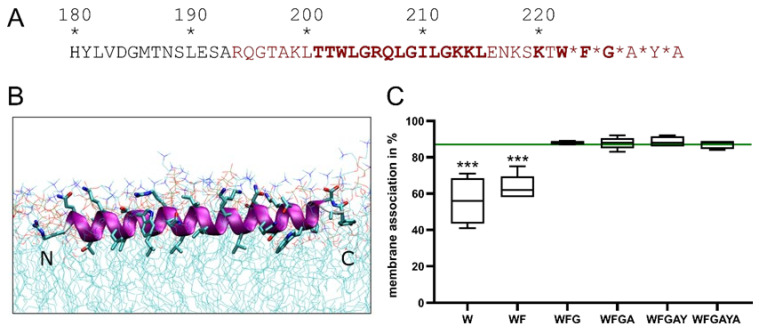
Features of the E^rns^ membrane anchor. (**A**) shows the carboxy-terminal sequence of E^rns^ from BVDV CP7. Amino acids in bold are those that were found to have no water accessibility in peptides in bicelles. Colored in red is the sequence that was used for the molecular dynamics simulation leading to the membrane anchor model shown in (**B**). The simulation was done for E^rns^ residues Arg194 to Ala227 at 72 ns in an explicit DMPC membrane. The protein shows a strong helical fold and lies slightly inclined in the hydrophobic region of the membrane just beneath the lipid head groups (see [16] for further information). Location of N and C terminus are indicated. (**C**) Percentage of E^rns^ that is membrane associated. Recombinantly expressed E^rns^ mutants that were successively shorted by a single amino acid from the carboxy-terminus (see stars that indicate the different stop sites in (**A**) were analyzed for their membrane association). Asterisks denote significant differences from the wt protein as tested with one-way ANOVA and a Dunnett’s post-hoc test with three stars representing *p* ≤ 0.001. Already the loss of four amino acids from the carboxy-terminus leads to a significant loss of membrane association.

E^rns^ is a special case among viral envelope proteins. It is an essential component of the virus particle, but is also secreted from infected cells and can be found in cell culture supernatant and the blood of infected animals [13,20]. It is membrane anchored, but does not have a transmembrane anchor. A relatively long stretch of the carboxy-terminus (Amino Acids 167–227) is implicated in the membrane association (Figure 1). Moreover, the carboxy-terminus of the protein alone is able to bind other proteins to membranes [36]. Shorter parts of the carboxy-terminus are less effective in conferring membrane association [36]. The complete sequence associated with membrane binding could theoretically be folded into an amphipathic helix. Alanine insertions (that would distort the amphipathic character of the helix) in this region severely impact membrane association [14]. A peptide corresponding to this sequence shows helix content of up to 80% in membrane mimicking environments [16,37]. Structural analyses using NMR and circular dichroism (CD) analyses as well as molecular dynamics analyses confirmed the helical character for at least the core region of this stretch (Figure 1B) [16]. Furthermore, these analyses indicated that the helix has a slightly tilted orientation. Proton exchange rates of peptides in bicelles indicated that a core part of the helix (T201 to K215) is completely submerged in the membrane or so strongly fixed in a rigid helical structure that proton exchanges with water are prevented. This finding points at absence of water accessibility for these residues [16]. This partial immersion fits nicely with the observation that E^rns^ binding to the membrane is stronger than for peripheral membrane proteins, but less strong than that observed for transmembrane proteins [36]. 

Early topology analyses using selective membrane permeabilization combined with immunofluorescence had already indicated that the protein is completely kept inside the ER lumen and ER membranes with no part accessible on the cytosolic face of the membrane. This result was confirmed by employing proteinase K protection assays [14]. These observations gave the first hints of the described in plane topology as the only adopted topology and further confirmed by the fact that CD spectra of peptides corresponding to the carboxy-terminus did not show any transmembrane alignment even at high concentrations [16]. Moreover, in contrast to other amphipathic helices, high concentrations of the corresponding peptides did not induce pore formation [16].

The last few amino acids in the carboxy-terminal part of the protein are structurally flexible, but submerged in the membrane [16] and have a high impact on membrane association of the complete protein (Figure 1C). The loss of the last four amino acids leads to a significantly reduced membrane association. They also play an important role in the E^rns^-E1 processing as discussed in the next part. 

### 3.2. E^rns^-E1 Processing

#### 3.2.1. The E^rns^/E1 Site Is Cleaved by Signal Peptidase

**Figure 2:**
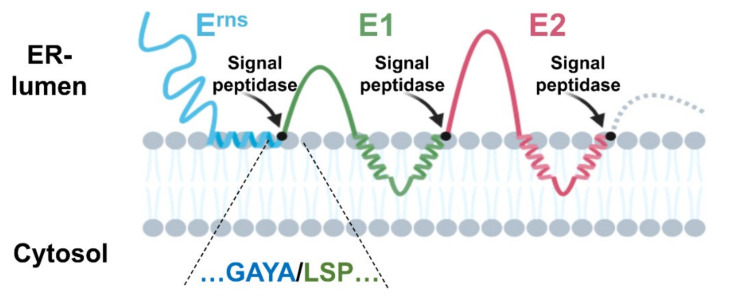
Processing of the pestivirus glycoprotein precursor. Schematic representation of the precursor with the three pestiviral glycoproteins in their precleavage topology (further details in [57]). The SP cleavage sites are indicated by black dots. For the E^rns^/E1 site, the flanking sequences are given below the scheme using the colors of the protein representations. The amino acid sequence is derived from CSFV Alfort/Tübingen.

Pestiviruses are typical positive strand RNA viruses with a genome encompassing a single long open reading frame [2,38]. Translation of the genomic RNA leads to a hypothetical polyprotein that is processed by cellular and viral proteases to give rise to the mature viral proteins. As pestiviruses are enveloped the translocation of the surface proteins has to be achieved during the translation process. A standard solution to this problem found in related viruses like HCV is to use an internal signal sequence located upstream of the amino-terminus of the first envelope protein for initiation of translocation [39,40]. Due to the combination of stop transfer and signal sequences in the polyprotein downstream of the initial signal sequence, a hypothetical multi membrane spanning structure is generated that is processed proteolytically (Figure 2). After cleavage, the combined membrane anchor/signal sequences stay at the carboxy-termini of the envelope proteins and serve as their membrane anchor. Finally, a stop transfer sequence enters the translocon and terminates the translocation. The cleavage steps necessary for release of the mature proteins are executed by cellular proteases, mainly signal peptidase (SP) (in pesti- and hepaci-viruses only one step is catalyzed by signal peptide peptidase, SPP) [41,42,43,44,45,46]. SP is a well characterized evolutionary highly conserved enzyme [47,48,49,50]. SP substrates usually display a three-domain design with a positively charged amino-terminal (n-) region, a central hydrophobic (h-) region forming an α-helical transmembrane sequence, and, downstream thereof, a more hydrophilic part (c-region) containing the actual cleavage site [31,32,33,34]. All of the SP cleavage sites in the pestivirus polyprotein conform to this pattern [3,10,35,36] except for the E^rns^/E1 site. Due to the atypical membrane anchor of E^rns^ the latter site is lacking the h-region, but comprises the carboxy-terminal amphipathic helix of E^rns^ associated in plane with the lipid bilayer surface on the luminal side of the ER membrane. The utmost carboxy-terminus of E^rns^ consists of a tripeptide with a terminal von Heijne cleavage motif, A-(Y/N/H)-A. Even though this carboxy-terminal sequence pointed at a cleavage of the E^rns^-E1 precursor by SP the completely different topology of the cleavage site sequence raised the question, whether SP indeed processes this site. 

Signal peptides, the regular substrates of SP, show a rather high degree of variation both with regard to the length and sequence of the n-, h- and c-regions [51,52,53]. This could give the impression that SP displays a high flexibility with regard to the structure of its substrate as long as the general scheme and the −1, −3 rule are obeyed. Some of the typical features of a signal peptide might even be only important for the initiation of translocation and not for its acceptance as a substrate by SP. In an oversimplified view, one could regard only the c-region, which interacts with the protease, as being responsible for substrate recognition. However, in reality SP shows a high degree of substrate specificity [51,54,55,56]. One important point in this context concerns the presentation of the cleavage site at a position accessible by SP because artificial lengthening of the h-region of a signal peptide prevents SP cleavage (67). Moreover, the alpha-helical conformation of the h-region must not extend into the carboxy-terminal part of the c-region, to ensure that the −1,−3 residues are located in a region of extended conformation (66,68,69). Thus, a parameter for substrate recognition should be the correct presentation of a suitable cleavage site in a given ‘cleavage space’ close to the membrane surface. It is, therefore, obvious that a variety of parameters are crucial to define whether a certain sequence represents an SP substrate. One of these parameters could also be the context of substrate and protease with the translocon. The different requirements defining a von Heijne motif as an SP cleavage site seem to almost exclude the E^rns^/E1 site as a SP substrate since both biochemical features and determined membrane topology are contradicting the various structural demands and defined interaction of this sequence with the Sec61 complex seems highly unlikely. 

To test whether SP is responsible for processing of E^rns^-E1, we first analyzed whether the cleavage occurs in the ER or another compartment [18]. In vitro translation experiments revealed that processing is dependent on the presence of microsomal membranes and thus should not occur in the cytoplasm. Treatment of cells expressing E^rns^-E1 with Brefeldin A (BFA), which blocks the ER to the Golgi transport [58,59], did not prevent the processing step [18]. This result supported the hypothesis that the processing step occurs in the ER but since BFA treatment leads to redistribution of Golgi components into the ER, involvement of Golgi proteins could not be excluded. If Golgi played a role it would have been restricted to cisGolgi components since endoglycosidase H (Endo H) treatment proved the absence of complex carbohydrates from E^rns^ and E^rns^-E1 expressed in BFA treated cells. Thus, E^rns^-E1 processing could be localized to the ER or cisGolgi compartment [18].

Mutagenesis analyses showed that the −3 and −1 positions of the E^rns^-E1 cleavage site (the alanine residues in the A-(Y/N/H)-A motif mentioned above) are crucial for processing as would have to be expected for a SP cleavage site. However, replacement of one of the As did not only prevent cleavage when one of the known bulky blocking residues like leucine was introduced but also rather small amino acids like cysteine at −1 or glycine at −3 interfered strongly with SP activity [18]. These results made obvious that sequence requirements exceeding those known for a typical SP cleavage site have to be fulfilled for this cleavage site. Moreover, deletion mutants and a variant with a single alanine insertion 36 residues upstream of the cleavage site showed considerably reduced processing of the precursor. This was most likely a consequence of disturbing membrane binding by the carboxy-terminal amphipathic helix of E^rns^ thereby impairing also the contact of the cleavage site with SP [18]. Surprisingly, also inhibition of protein glycosylation was able to prevent E^rns^-E1 processing although this manipulation does not directly affect the carboxy-terminus of the protein. Taken together, all these findings point at structural requirements for proper interaction between enzyme and substrate that are not known for SP so that the question whether this protease is indeed active in E^rns^-E1 processing was still not answered by these analyses. However, purified SP can execute a specific posttranslational cleavage of a substrate in in vitro assays [49,50,60,61] and this activity can be blocked by a specific SP inhibitor [62,63,64,65]. We were able to show that also E^rns^-E1 cleavage could be blocked by this inhibitor [18]. As a final conclusion, these data demonstrated that the E^rns^-E1 precursor represents a substrate for cleavage by SP, though a very unusual one since the structure of the E^rns^-E1 cleavage site most likely does not allow a regular interaction of substrate and SP with the translocon. This interaction was proposed as one requirement and explanation for the specificity of SP for a small number of sites among a huge number of sequences fulfilling the von Heijne −3/−1 rule [51,66,67,68,69,70,71,72,73]. Somehow, the strange arrangement with the amphipathic helix that usually attaches in plane to the membrane surface seems to be able to mimic the crucial structural features of standard SP substrates.

#### 3.2.2. Importance of Specific Amphipathic Helix Residues for E^rns^-E1 Processing 

**Figure 3:**
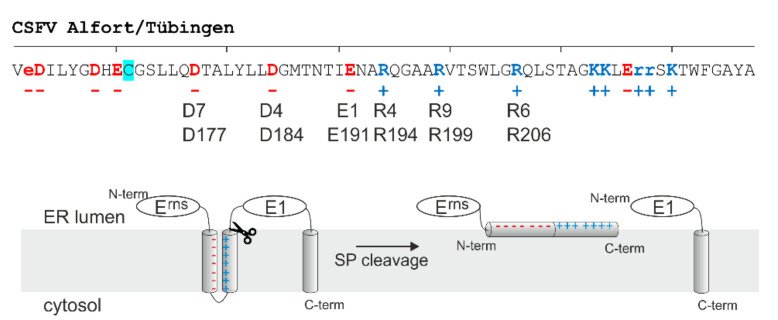
Scheme presenting the E^rns^ charge zipper hypothesis. The upper part shows the carboxy-terminal amino acid sequence of E^rns^ (sequence CSFV Alfort/Tübingen). Charged residues are shown in red (negative charge, indicated also by “-“) or blue (positive charge, indicated also by “+”). Cysteine 171, which is responsible for E^rns^ homodimerization, is highlighted with a turquoise background. Above the amino acid sequence, a scale with 10-residue calibration starting with glutamic acid at Position 170 is shown. Below the amino acid sequence, the net charges of the residues are given. The designations of the amino acids mutated in the studies presented here are given with the short forms used especially in the figures and the full number as used in the text: D7 = Asp177, D4 = Asp184, E1 = Glu191, R4 = Arg194, R9 = Arg199, and R6 = Arg206. The bottom part shows a scheme summarizing the charge zipper theory proposed to be involved in processing the E^rns^-E1 precursor. The charge zipper allows the initial formation of a hairpin structure inserted into the membrane, thereby forming a suitable substrate for SP cleavage. After processing, the E^rns^ carboxy-terminus is proposed to re-orientate and form an amphipathic helix binding in plane to the membrane surface as experimentally determined for the mature protein [16]. The scheme was based on the original presentation of the hypothesis [78] but modified according to recently published data on the role of E1 for the processing step [19].

As described in the previous chapter, deletions or insertions in the amphipathic helix influence E^rns^-E1 processing [18] so that one can speculate that the correct structure of the helix is important. This may simply be because optimal folding is necessary for membrane binding of this sequence, which certainly is a prerequisite for SP cleavage. Usually, membrane binding of an amphipathic helix is regarded as a process governed by the hydrophobic face of the helix. This face is certainly the key player for establishment of lipid contact, but also the residues on the hydrophilic side are important determinants for establishment of the structure [74,75,76,77]. More detailed analysis of the sequence of the E^rns^ carboxy-terminus revealed a conserved distribution of charged amino acids on the hydrophilic side of the amphipathic helix [78]. The charges of these residues are distributed in a way that opposite charges are located on both sides of a mirror axis. In consequence these residues can establish up to eight salt bridges when the helix folds into a hairpin structure (Figure 3). 

Similar arrangements were found in a group of other proteins, for which (reversible) establishment of the salt bridges was demonstrated with biophysical techniques. Moreover, establishment of these salt bridge stabilized structures was found to be functionally important as for the bacterial protein TatA, for which such salt bridges are responsible for folding and self-assembly of an oligomeric pore structure [78,79]. Because of the arrangement of the charges in the protein one can hypothesize a step-by-step establishment of salt bridges starting at one point so that such groups of opposite charges were named “charge zipper”. In the case of E^rns^, the (intermediate) formation of a hairpin structure stabilized by up to eight salt bridges was an attractive model in the context of the carboxy-terminal SP cleavage since the zipped structure of the membrane anchor could re-orientate the helix from the described in plane localization parallel to the membrane surface [16] to a hairpin transmembrane conformation, which would be very similar to the usual SP substrate.

**Figure 4:**
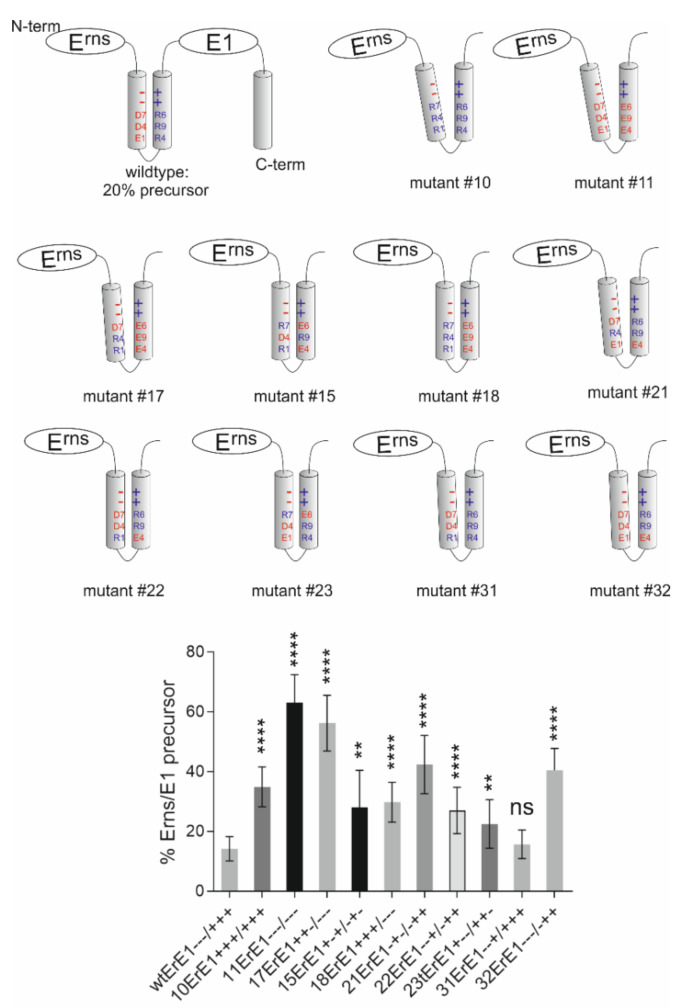
Effect of mutations of the conserved inner six charged residues in the E^rns^ carboxy-terminal helix on E^rns^-E1 processing. The upper part shows a schematic presentation of the mutants in the putative charge zipper region of E^rns^. Amino acids are as described above (D7 = Asp177, D4 = Asp184, E1 = Glu191, R4 = Arg194, R9 = Arg199, and R6 = Arg206). Red color symbolizes negative charge (also indicated by “-“), and blue color positive charge (also indicated by “+”). The tilted helices shown for mutants 10 ErE1, 11 ErE1, 17 ErE1, 21 ErE1 and 32 ErE1 indicate the different degree of repulsion induced by the charge changes. The bar diagram summarizes the results of immunoprecipitation experiments after expression of E^rns^-E1 with the mutations shown above. The numbers of the constructs together with the charge distribution patterns are indicated at the X-axis. The bars represent the percentage of uncleaved E^rns^-E1 precursor determined in at least three independent experiments. The calculation principle is described in Materials and Methods section. Error bars are indicated and the *p*-Values for the results determined for the mutants with respect to the wt E^rns^-E1 are indicated by asterisks. Significant differences of the E^rns^-E1 processing rate were observed for constructs in 10ErE1 and 11ErE1 (*p*-Value < 0.0001), 15ErE1 (*p*-Value = 0.0022), 17ErE1 (*p*-Value < 0.0001), 18ErE1 (*p*-Value < 0.0001), 0.21 ErE1 (*p*-Value < 0.0001), 22ErE1 (*p*-Value < 0.0001), 23ErE1 (*p*-Value = 0.0093), and 32ErE1 (*p*-Value < 0.0001), whereas 30ErE1 gave no significant difference (Ns = not significant). *p*-Value ranges: * ** = <0.01 and **** < 0.0001.

We have challenged the charge zipper hypothesis in elaborate mutagenesis studies based on an expression construct coding for E^rns^-E1 of CSFV [80]. For maximal effects, we started with mutations exchanging amino acids for residues with opposite charges because this would not only destroy the respective salt bridge but lead to repulsion. Moreover, this approach allowed the restoration of the ability to form salt bridges by reversing the charges on both sides of the mirror axis. Figure 4 shows some examples of the tested mutants. As can be seen in the bar diagram some mutants containing replacements with opposite charges showed severely reduced E^rns^-E1 processing rates. We observed a tendency of increased effects correlated with the number of introduced mutations, but this was not a general result. Importantly, the effect of the exchanges was clearly dependent on their position in the helix with R194E representing the mutation with the strongest contribution to processing impairment. In general, replacement of the positively charged amino acids by acidic amino acids resulted in stronger effects than mutations affecting the negatively charged residues. This is especially obvious for the construct with the three inner arginines replaced by glutamic acid (mutant 11ErE1) displaying a much higher reduction of cleavage compared with the reciprocal mutation (inner three acidic residues replaced by arginine, mutant 10ErE1). These results somewhat contradicted the charge zipper hypothesis since the latter two constructs from a charge zipper aspect should be equal. Even more obvious, the E^rns^-E1 cleavage rate did not correlate with the number of salt bridges a mutant protein could establish. 

This could be seen when the processing rate of, e.g., construct 17ErE1 was compared with that of mutants 10ErE1 or 11ErE1. While the proteins derived from the latter two have lost the ability to establish all three inner salt bridges, the former codes for a polypeptide with conservation of two possible inner salt bridges because of partially reciprocal changes on both sides of the putative charge zipper. Nevertheless, the loss of processing efficiency of mutant 17ErE1 is in the same range as mutant 11ErE1 and even higher than for 10ErE1. In addition, the full reciprocal change allowing three inner salt bridges as in constructs 15ErE1 or 18ErE1 does only partially restore E^rns^-E1 processing (Figure 2). There are a lot more examples where the determined E^rns^-E1 cleavage efficiency differs from the effects expected in consideration of the charge zipper hypothesis. Thus, it can be concluded that the charged residues in the E^rns^ amphipathic helix play a role in E^rns^-E1 processing but a clear indication for a charge zipper based process in connection with this cleavage could not be found [80].

#### 3.2.3. The Role of E1 and E2 in E^rns^-E1 Cleavage

As pointed out above, processing of the E^rns^-E1 precursor is executed by SP and the efficiency of this action is strongly dependent on the integrity of the E^rns^ membrane anchor including the presence of the conserved charged residues located on the hydrophilic face of the amphipathic helix. During the initial analyses of E^rns^-E1 processing the influence of the E1 moiety was not investigated. The main reason for this strategy was the expected absence of any significant influence of this part of the precursor on the processing step since SP is known to be very flexible with regard to the sequence downstream of the cleavage site. However, the unusual structure of the cleavage region, the requirements exceeding those of regular cleavage sites and the results of analyses focused on side aspects of the processing scheme indicated that E1 could be important. We, therefore, started an investigation to evaluate the role of E1.

The observation that E^rns^-E1 represents a rather stable product of pestiviral polyprotein processing indicated that SP cleavage at the unusual E^rns^/E1 site is somehow delayed compared to other SP cleavages. One logical explanation would be that folding and membrane association of the amphipathic helix needs some extra time and, therefore, cannot occur in the context of the co-translationary translocation of the protein. Since cleavage at the E1/E2 site precedes E^rns^-E1 processing [19] the carboxy-terminal membrane anchor of E1 is obviously the only structure able to ensure association of the precursor to the lipid bilayer. We, therefore, speculated that the E1 membrane anchor could be necessary to keep the precursor in close vicinity to SP and give time for folding and membrane insertion of the amphipathic helix as a prerequisite for proteolytic cleavage. To verify this hypothesis, we analyzed the effect of increasing carboxy-terminal truncation of E1 on E^rns^-E1 processing. Cleavage of the truncated proteins was severely impaired and the precursors were strongly secreted [19]. This finding was in agreement with our hypothesis, which would also imply that the caboxyterminal ~30 amino acids contain the E1 membrane anchor and thus play the crucial role in this process when membrane anchoring is the important feature. We, therefore, established constructs coding for proteins with different internal deletions preserving the last 30 residues of E1. Interestingly, these proteins again showed strongly reduced processing even though the presence of the E1 carboxy-terminus prevented increased secretion ([19] and Section 3.6.2). Thus, processing at the E^rns^/E1 site, and maybe also the establishment of a stable membrane contact of the E^rns^ amphipathic helix is not only a matter of time and membrane association of the precursor protein. The defect induced by the truncation could not be compensated by expression of full length E1 in trans so that the most logical explanation of our findings is that E1 has to fold properly in order to generate a cleavable structure at the E^rns^/E1 site and that this fold is very sensitive to changes affecting E1. 

It is important to notice that E1 seems to have an active role in promoting processing instead of just hindering cleavage when truncated since also very short E1 derived extensions are not cleaved off efficiently. This effect is not restricted to E1 sequences: also a c-Myc that can hardly be imagined to impose a structural block on the cleavage site is not cleaved off when fused to the E^rns^ carboxy-terminus [19]. This is somewhat reminiscent of a finding published for gp160 of human immunodeficiency virus (HIV) [51]. The signal peptide of the glycoprotein precursor is removed only after completion of translation and correct folding of the subunit gp120. This ordered procedure is a consequence of special features of the signal sequence, in which the h and c regions overlap. As a result, the SP cleavage site is located inside of the lipid phase and, thus, not accessible for the protease. The gp120 stays tethered to the membrane via its amino-terminal signal sequence and thus has time for correct folding. Interaction of the proteins’ amino- and carboxy-termini in a late folding step was proposed to induce a conformational change in the amino-terminal region leading to a cleavable α-helical structure. As in our case, carboxy-terminally truncated gp120 showed only poor signal sequence cleavage, which also in the wt configuration occurs delayed and requires (nearly) complete folding of the protein [51].

Another case of delayed SP cleavage is known for members of the genus *Flavivirus* where SP cleavage generating the amino-terminus of prM can only occur when the viral serine protease has removed the signal sequence from the carboxy-terminus of the capsid protein C [73,81,82]. This ordered process is dependent on specific features of the signal peptide and the downstream prM sequence [83]. The delay of SP cleavage is functionally important as optimization of the processing in Yellow Fever virus was lethal [84].

The expression of E^rns^-E1 with increasing internal deletions in E1 demonstrated that processing at the E^rns^/E1 site and intracellular retention of the protein are not directly linked [19]. Interestingly, the correct amino-terminal sequence of E1 is obviously not essential for efficient cleavage since even exchange of the conserved residues in this part of E1 had no significant impact on processing but impaired recovery of viable viruses. Thus, the (delayed) cleavage of E^rns^-E1 seems not to be dependent on the utmost primary sequence downstream of the cleavage site [19].

It, therefore, can be concluded that a certain fold at the cleavage site, maybe together with interaction with the E1 carboxy-terminus, is necessary for efficient E^rns^-E1 cleavage. Truncated E1 sequences prevent both membrane binding and adaptation of a cleavable structure whereas a non-related sequence like the c-Myc tag allows membrane binding but still prevents the cleavage. This observation again supports the conclusion that full length E1 actively promotes processing. The available data lead to a processing scheme for the amino-terminal third of the pestivirus polyprotein with autoprotelytic removal of N^pro^ as a first step, followed by the cleavage at the C/E^rns^ site during translocation of the glycoprotein precursor. In contrast, a rather stable E^rns^-E1-E2 fusion protein is generated that is detectable in infected or transfected cells. Third step is cleavage of the E1/E2 site in this precursor generating E2 and the E^rns^-E1 fusion protein, both bound to the membrane via carboxy-terminal membrane anchors. Our results show that release of E2 has to occur in order to allow efficient processing (see below), for which correct folding of E1 is necessary [19]. The importance of the cleavage at the E1/E2 site as first step of the processing of the E^rns^-E1-E2 fusion protein could be due to a requirement of a free E1 carboxy-terminus, which according to our results re-orientates from a banana-like intermediate structure to a transmembrane configuration [57]. The reorientation could be a prerequisite for the (correct) interaction with the amino-terminal cleavage site region in analogy to HIV gp160 [51]. After release of E2, E1 is able to establish a correct structure, which initiates membrane binding of the E^rns^ amphipathic helix and cleavage at the E^rns^/E1 site. Our earlier data showed that the conformation of the amphipathic helix is also very important for processing and intracellular retention of E^rns^ [14,18]. Thus, the correct conformation of the proteins on both sides of the cleavage site and maybe also its interaction with the E1 carboxy-terminus is a crucial prerequisite for processing. This finding proves that SP can accept this unusual substrate only under very restricted conditions.

It is not known whether the delayed processing at the E^rns^/E1 site is functionally important. One possible scenario could be that processing of E^rns^-E1 is prevented before both proteins have established their final 3D structure. This could ensure proper folding and shielding of crucial structures like a putative membrane fusion domain to prevent their premature activation. Such strategies were elucidated for many viral fusion proteins (see [85,86,87,88] for recent reviews). However, it is important to notice that the protein responsible for fusion in pestiviruses is still not known. E^rns^ is obviously dispensable for pestivirus membrane fusion [3,4], whereas fusion activity of E1 has been hypothesized but so far not proven [3,4,89,90,91,92]. A process of reciprocal chaperoning as described for HCV E1 and E2 [93,94] could also be necessary for E^rns^-E1 folding with the integrity of the E^rns^-E1 precursor representing a prerequisite for this mechanism. However, E^rns^ expressed in the absence of any other viral protein is able to bind to membranes and achieve efficient retention in the ER [17,37]. Moreover, mutants with large deletions affecting either the E^rns^ or the E1 coding region of the pestiviral genome can be rescued on complementing cell lines [46,95,96]. Thus, the generation of an E^rns^-E1 fusion protein anchored in the membrane via the E1 carboxy-terminus cannot be a prerequisite for generation of a membrane bound E^rns^ or the production of infectious pestivirus particles. However, a bicistronic construct expressing E^rns^ from a second ORF resulted in retarded virus growth compared with a similar construct, in which the second ORF coded for E^rns^-E1. This finding argues in favor of an advantage for a virus being able to express an E^rns^-E1 fusion protein [46]. Thus, a functional role of the delayed and coordinated E^rns^-E1 processing could exist.

### 3.3. Intracellular Retention and Localization of E^rns^

**Figure 5:**
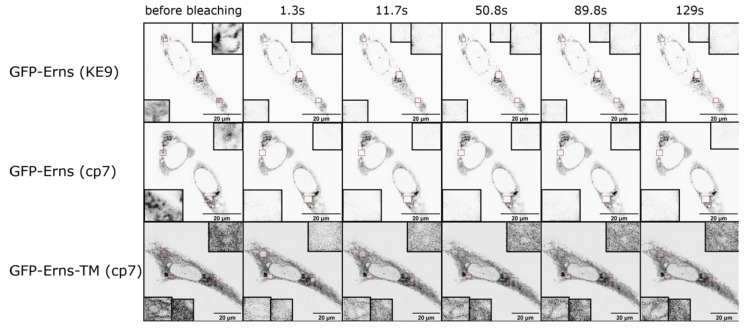
E^rns^ is statically retained in the ER. BHK-21 cells were transfected with plasmids coding for different GFP tagged variants of E^rns^, namely E^rns^ of BVDV CP7 (GFP-E^rns^ CP7), E^rns^ of BVDV KE9 (GFP-E^rns^ KE9) and E^rns^ of BVDV CP7, in which the membrane anchor had been replaced by a hydrophobic sequence (GFP-E^rns^-TM CP7). The transfected cells were used in FRAP experiments. Shown are images directly before the bleaching and at the indicated time intervals after bleaching. The bleached areas are magnified in the insets in the corners.

Pestiviruses bud intracellularly from the ER membrane [97]. The viral structural proteins need to be retained near the budding site and since E^rns^ is a viral structural protein [22,98] this holds true for E^rns^. Thus, while E^rns^ is secreted to a certain amount [13], the protein is also retained in the cell [14,36,37,99]. In fact, similar to the envelope proteins of HCV [100,101,102,103] E^rns^ [37], E1 [57] and E2 [103,104] are located in the ER of infected cells, as well as when they are expressed recombinantly. Retention is independent of other viral proteins as E^rns^ alone expressed recombinantly is readily retained within the cell [14,37]. Mapping of the retention signal shows that intracellular retention and ER localization is due to the E^rns^ carboxy-terminus [37], thus its membrane anchor. In this, the protein shows great similarity to E1 and E2 [57,103] and the envelope proteins of hepaciviruses and flaviviruses that are all retained by their transmembrane domains [100,101,102,105], even though its membrane anchor is fundamentally different. The membrane anchor of E^rns^ alone is sufficient to retain another protein (CD72) in the cell [37]. Distortion of the amphipathic helix leads to loss of the retention ability. A closer mapping identified amino acids that are crucial for the retention. To differentiate between the effect on membrane association and retention the former was determined using the wt protein and measuring the amount of secreted protein. The latter was measured in CD72-fusion proteins, that remain membrane bound due to the CD72 membrane anchor. L183, I190 and L208 have an effect on retention in the CSFV Alfort/Tübingen E^rns^ [37]. They also play a role overall in membrane association, but interestingly, the retention is much more sensitive to mutations at these positions compared to membrane association. Retention can be achieved by several different strategies. Retention through KDEL (for soluble proteins) or KKXX (for transmembrane proteins) is achieved through a retrieval process, by which the proteins travel on the secretory pathway and are brought back to the ER from the ERGIC through interaction with ER-retrieval receptors and COPI-mediated transport. Another possible mechanism of retention is a static retention where the proteins do not travel along the secretory pathway. An example of this kind of retention is the retention of the luminal ER protein TorsinA, which contains an amino-terminal amphipathic helix. This structure binds it to ER membranes from the luminal side and is responsible for the retention by excluding the protein from ER exit sites [106]. The retention of E^rns^ was analyzed in live cell imaging using GFP-E^rns^ fusion proteins in FRAP experiments. Similar to TorsinA (as an example for a monotopic ER protein) and HCV E1 (as an example for a viral surface protein) [101], BVDV E^rns^ shows static retention in the ER. This could be seen with GFP-tagged E^rns^. GFP-tagged E^rns^ did not refill bleached regions in FRAP experiments in the selected time frame, which would be expected if the protein cycles constantly on the secretory pathway (Figure 5). The control, an E^rns^ with an artificial transmembrane anchor, shows exactly the phenotype expected from a cycling protein (Figure 5).

### 3.4. Dimerization of E^rns^

As mentioned already above, E^rns^ forms homodimers that are covalently linked via cysteine 171. The dimers are found in infected cells, cells transiently expressing E^rns^ in the absence of any other viral protein and in virions [7,8,22]. Interestingly, our experiments showed that formation of stable E^rns^ dimers is not necessary for virus viability. Moreover, the ability to establish these homodimers seems to represent no significant advantage for the viruses during tissue culture propagation [7]. However, cysteine 171 in E^rns^ has been conserved during the evolution of pestiviruses. The vast majority of pestivirus isolates contain this codon and should, therefore, be able to express E^rns^ homodimers. Only very few of the available pestivirus sequences display another amino acid at this position [7]. The conservation of C171 in pestiviruses points towards an advantage for viruses that can express E^rns^ homodimers during replication in their natural hosts. Indeed, our animal studies revealed considerable attenuation of CSFV mutants generated via reverse genetics with a deletion or mutation of cysteine 171 [7,8]. We proved that such manipulations prevent E^rns^ dimer formation but do not abrogate its RNase activity [7]. With regard to the symptoms of disease recorded during the animal studies the E^rns^ dimer-negative CSF viruses behaved very similar to virus mutants with inactivated E^rns^ RNase. Compared to the wt virus, viremia was significantly reduced in animals infected with homodimer- or RNase-negative viruses. Since both types of mutants induced in the pigs significant levels of neutralizing antibodies the attenuation was most likely not due to dramatically hampered virus replication in the host animals [7]. Thus, it is tempting to speculate on a specific mechanism of interference with the innate immune system of the host, which is dependent on both the RNase function and the homodimerization of E^rns^. In any case, this idea would imply that reverting to a dimer positive phenotype should provide a significant advantage, so that such revertants would finally overgrow the original mutants and could be identified in the animals. In fact, we were able to isolate (pseudo)revertants from pigs infected with C171 mutants, and thus, strongly support the idea of a specific role of E^rns^ homodimers. Reversion was detected in animals that had been infected with a point mutant V-C171F whereas the deletion mutant V-C171d did not yield revertants [8]. The latter had to be expected since a deletion cannot be reverted as easily as a single amino acid exchange. 

As outlined above the detection of true revertants is not surprising when the change results in enhanced growth. A surprising finding in our analyses was the detection of pseudorevertants that preserved the original change of C171 but contained a serine to cysteine change quite far away from this position. Importantly, such mutants were not only found in animals infected with the C171 deletion mutant not able to truly revert but also in animals inoculated with the point mutant V-C171F [8]. This finding showed that the pseudoreversion represented a real alternative to reversion. However, we never found pseudoreversion of the V-C171S mutant, indicating that the amino acid at Position 171 has at least an important influence on the tendency for reversion or pseudoreversion. Apparently, the similar biochemical character of serine and cysteine reduces not only the pressure towards reversion but also to pseudoreversion. As V-C171S also shows a growth reduction that is apparently compensated when S209 is converted to C, this finding is interesting but cannot be explained at the moment.

We were able to prove that the S209C mutation restored the ability to generate E^rns^ homodimers in all tested mutants lacking C171. The monomer/dimer ratio of proteins with C171 or C209 was very similar demonstrating that cysteines at Position 209 are able to form disulfide bonds without significant structural or steric hindrance. This is somewhat surprising since amino acid 209 is located within the central part of the amphipathic helix supposed to be bound to and maybe even submerged in the lipid bilayer [8,14,16]. In contrast, the regular cysteine at Position 171 is found rather close to the amino-terminal end of the membrane anchor region, but not in contact to the membrane. Comparing the growth rates of the recovered viruses revealed that those with dimers formed via C209 had per se no disadvantage since the C171S/S209C mutant grew almost like wt. Impaired growth rates determined for the other mutants seemed to be independent of the exchange at Position 209 but connected with the type of change at Position 171 (more significantly reduced growth rates for the C171F and C171Δ mutations). Even a double mutant with cysteines at Positions 171 and 209 (V-C/SC) replicated with wt efficiency despite the fact that it expressed oligomeric forms of E^rns^ [8]. It has been published that pestiviruses need not only a pH shift but probably also a reduction step for infection [27]. It, therefore, might be that E^rns^ dimers or even oligomers are reduced to monomers during infection, and, therefore, the presence of dimers or oligomers in the virion has no influence on virus performance in tissue culture. 

According to our NMR CLEANEX results and computer aided structure predictions, Residue 209 is part of a long stretch of amino acids that seem to be buried in the lipid bilayer [16]. It is, therefore, not clear why pseudoreversion has only been found at this position. It might be, that serine 209 is one of very few residues in the highly conserved membrane anchor that allow substitutions. A variety of independent CSFV sequences show 100% conservation of the sequence from C171 to the carboxy-terminal end of E^rns^. Variants with one exchange in the membrane anchor often display the change at position 209 (mostly glycine or arginine). Since further somewhat variable residues are found in direct vicinity to 209 (Positions 210, 211) it might be that this region provides some flexibility. However, it must also be kept in mind that the exchange of cysteine for serine is highly conservative with regard to the biochemistry of the protein. Alternatively, also structural constraints restricting the possible positions for dimer formation have to be taken into account as reason for the predominance of pseudoreversion at Position 209. This interesting question can only be answered by further experimental work, which should also include analyses allowing conclusions on the so far unknown general role of the E^rns^ membrane anchor for the generation of E^rns^ homodimers.

In the light of our hypothesis of a direct connection between E^rns^ homodimer formation and pestivirus virulence the most interesting aspect of the identified pseudorevertants was the possibility to test in animal studies whether restoration of dimerization via the S209C mutation also restores virus virulence despite the preservation of the original changes at Position 171. Indeed, animals inoculated with the pseudorevertants showed higher clinical scores, slightly stronger initial drop of WBC counts, and higher viral loads compared with the original dimerization negative mutants [8]. It was not surprising, that the pseudorevertant did not regain the virulence of the wt virus since the introduction of two exchanges into a highly conserved and functionally important sequence element can be expected to have effects on the fitness of the virus, even when the primary functional defect is eliminated. Because of the limited number of animals available for such studies these results do not meet all criteria for statistical significance but the results clearly support the conclusion of an increase in virulence in consequence of restoration of E^rns^ dimer formation. 

Taken together, our analyses have provided compelling evidence that homodimerization of E^rns^ is important for the virulence of CSFV and most likely also pestiviruses in general. The molecular mechanism behind this finding is still unclear. Dimerization was found to be crucial for the biological functions but not enzymatic activity of another RNase, named bovine seminal (BS) RNase. This RNase is closely related to RNaseA but is secreted from the producing cells as a dimer and then internalized by target cells. It has strongly immunosuppressive activity, which is most likely due to the fact that the dimeric form of the enzyme is not efficiently blocked by intracellular RNase inhibitors, so that internalized BS RNase provokes cell death in consequence of degradation of cellular RNA [107,108,109,110,111]. It is tempting to speculate that also in pestiviruses the ability to express E^rns^ RNase homodimers is important for the described immunosuppression by these viruses and that blocking dimerization prevents the RNase to become active at it’s so far unknown place of biological function. Thus, inactivation of the E^rns^ RNase activity and prevention of its homodimerization could result in equivalent effects, namely the attenuation of the viruses.

### 3.5. E2 as a Further Player

**Figure 6:**
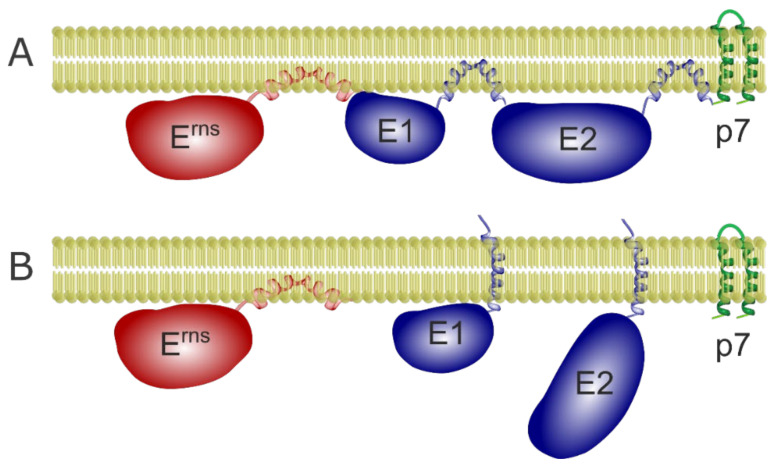
Schematic drawing of the envelope proteins and p7 before (**A**) and after (**B**) processing. Before cleavage the membrane regions of all three proteins start and end in the ER-lumen, with the E^rns^ membrane anchor in the more likely in plane configuration, but slightly tilted, and the other two most probably in a banana-like or hairpin conformation. After cleavage the E^rns^ membrane anchor stays in plane in the membrane while E2 and E1 adopt single span transmembrane conformations [14,57,103].

It has been reported that E^rns^ can form heterodimers with E2 and E2-p7 in infected MDBK cells [112]. E2 is the major envelope antigen in infection and the protein responsible for receptor binding [113]. It forms homodimers and heterodimers with E1. E2 is also retained in the ER and the retention is mostly due to an arginine in the E2 transmembrane domain [103,104]. Cleavage between E1 and E2 is fast and represents a prerequisite for E^rns^-E1 processing as outlined above [19]. In contrast, processing between E2 and p7 is slow leading to noticeable accumulation of E2-p7 precursor [114] and making the E1-E2 processing different than the E^rns^-E1 cleavage. E^rns^, E1 and E2 are retained in the ER and contain their own retention signal [37,57,103,104]. In contrast to E^rns^, mature E1 and E2 adopt a single span transmembrane topology (Figure 6), as seen by selective permeabilization and immunofluorescence [57,103]. E2 and E1 interact through polar residues in the membrane region that for E2 are also important for the retention [4,103,104]. It has been proposed that retention of E1 is through the interaction with E2. The fact that some mutations in E2 that lead to loss of retention can be compensated through the interaction with E1 hint that this protein must also have its own retention mechanism [103]. We have proven recently that E1 has indeed its own retention signal and does not depend on the co-expression of E2 for its ER localization [57]. In this context, it is especially interesting that E2 uses the same amino acids for retention and interaction with E1. 

It has also been proposed that E2 might be responsible for the retention of E^rns^ in the cell and for the presence of E^rns^ on viral particles through heterodimer formation. The fact that E^rns^ has its own unique membrane anchor (see Section 3.1) and contains its own retention signal (see Section 3.5) clearly refutes this theory.

Both E1 and E2 adopt a single span transmembrane conformation in their mature form but adopt hairpin or banana conformations in the polyprotein so that their membrane anchor can act as a signal sequence for the following protein (Figure 6).

### 3.6. Secretion of E^rns^

#### 3.6.1. Importance of Specific Amphipathic Helix Residues for Secretion of E^rns^

As described above, the carboxy-terminus of E^rns^ plays an important role in processing at the E^rns^/E1 site of the pestiviral polyprotein [80]. This is achieved by folding of the sequence into the amphipathic helix and its binding to the lipid bilayer. Establishment of membrane contact can be hypothesized to coincide with helix formation, which arrangement would fit well with the observed processing delay. However, the amphipathic helix is also crucial for E^rns^ membrane anchoring and intracellular retention [2,14,16,17,37], and the proposed process of concerted folding/membrane binding and subsequent SP cleavage would assure highly efficient membrane anchoring of E^rns^. Our early work already proved that the integrity of the amphipathic helix is crucial for efficient membrane binding, since single residue insertions inducing a twist of approximately 110 degrees of the parts of the helix upstream versus downstream of the insertion site resulted in much higher E^rns^ secretion rates. The intensity of the effect was dependent on the site of insertion. This observation can be explained by a twist of large (insertion in the middle) or small (insertion close to the ends of the structure) areas of the helix [14]. 

**Figure 7:**
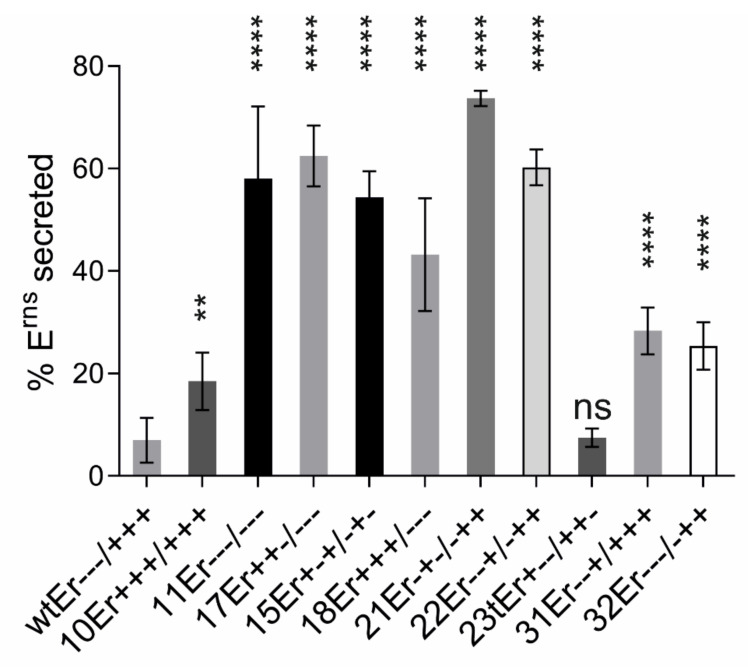
Charge exchange mutations in the membrane anchor can increase E^rns^ secretion. Results of immunoprecipitation experiments from extracts or supernatants of BHK-21 cells transiently expressing E^rns^ (from construct SE^rns^ [19]) or mutants thereof with exchanges affecting the charged residues in the E^rns^ carboxy-terminus. The radioactively labelled proteins were precipitated from the supernatant and cell extract with E^rns^ specific mab 24/16, treated with PNGase F and separated by SDS-PAGE. The diagram summarizes the results of at least three independent experiments quantified via phosphorimager analysis. The number of the individual construct together with the charge distribution in the E^rns^ carboxy-terminus are given at the X-axis. The bars represent the amount of secreted protein as percent of total recovered E^rns^. For calculation, the counts determined for extra- and intracellular E^rns^ were set to 100% expression product as basis for calculation of the secretion value. Error bars are indicated as well as the *p*-Value of E^rns^ mutants compared to E^rns^ wt with **** representing *p* < 0.0001, ** *p*= 0.002 for 10Er and ns = not significant for 23Er. “-“ indicates a negative, “+” a positive charge.

In addition to experiments aiming at investigation of the general role of the amphipathic character of the E^rns^ carboxy-terminus for membrane binding we also analyzed the importance of specific sequence patterns [80]. As a first step, we tested the influence of the above mentioned conserved charged residues on membrane binding and intracellular retention of E^rns^ expressed as a solitary protein without E1, and compared the secretion level with that of wt E^rns^. We tested mutants with one to seven exchanges with amino acids carrying the opposite charge to the original residue (Figure 3 and Figure 4). A selection of the results is shown in Figure 7. The presented constructs contain the same exchanges as those shown in Figure 4 for plasmids tested in the context of the processing of E^rns^-E1. All of these mutants show significantly increased E^rns^ secretion rates. Comparison with the processing analyses reveals that there are obvious differences with regard to the effects of individual changes on processing versus secretion indicating that the role of the amphipathic helix for these two processes is somewhat divers. It can be hypothesized that these two processes rely on at least partially different mechanisms. Interestingly, all exchanges of single amino acids with opposite charge had significant effects on E^rns^ secretion with at least doubling the amount of extracellular E^rns^ with only one exception, mutant D177R (mutant 29E+--/+++) (Figure 7 and [80]). It is obvious that the other alterations have quite variable effects with some mutants exhibiting only moderately increased secretion rates whereas strong increases up to ~75% of E^rns^ in the supernatant were detected for others. Most importantly, a strict dependency of the secretion rate on the number of exchanges was not observed, even though a tendency in this direction was obvious. As for the processing, compelling evidence for a charge zipper as relevant structure for membrane binding and retention of E^rns^ was not found. An interesting observation already made when analyzing E^rns^-E1 processing was the generally higher effect of mutations affecting the arginines downstream of the mirror axis. Except for the obvious high frequency of the mutation R194E in highly secreted mutants, a generally higher influence of individual residues on the retention/secretion ratio was not detected. Experiments with further mutations effecting Position 194 revealed that the absence of a negatively charged amino acid 194 was critical for both processing and retention, since introduction of Mutation R194A had only minor or no significant effects. 

#### 3.6.2. The Role of Carboxy-Terminal Extensions for Secretion of E^rns^

Sequences fused to the carboxy-terminus of E^rns^ led to significantly increased secretion of the protein. This was first recognized in our experiments with carboxy-terminally truncated E^rns^-E1 fusion proteins aiming at investigation of the influence of E1 on processing of the E^rns^-E1 precursor already described above (3.2.3). We analyzed the same truncated E^rns^-E1 constructs with regard to the amount of secreted proteins. For all E^rns^-E1 proteins truncated by 30 or more carboxy-terminal residues of E1 we found significant secretion that by far exceeded the level determined for wt E^rns^ without E1 sequences or a fusion protein composed of both full length E^rns^ and E1 [19]. An endoglycosidase H (EndoH) assay with proteins precipitated from cell lysates proved that this raised secretion rate was due to increased transport of the fusion proteins along the secretory pathway since the amounts of EndoH resistant products were much higher for the truncated proteins (6% for SE^rns^ alone versus 44% or 37% for the version with 84 residues of E1[SE^rns^-E1:84] or the version with 139 residues of E1 [SE^rns^-E1:139], respectively) [19]. Taken together, truncated E1 extensions fused to the E^rns^ carboxy-terminus not only impair E^rns^-E1 processing but also interfere with intracellular retention of the fusion protein despite the presence of the known E^rns^ retention signal [37].

This was even true for very short E1 extensions fused to E^rns^. Only an extension by one single amino acid did not lead to significantly increased secretion levels whereas the values determined for all other tested variants (E^rns^ with 3, 5, 7, 8, 9 or 10 residues of E1) were significantly above the level of the wt protein [19]. These results show that also very short E1-derived sequences fused to the E^rns^ carboxy-terminus are mostly not cleaved off but lead to increased secretion of the expressed proteins from the cells. 

However, not all sequences fused to the E^rns^ carboxy-terminus lead to strongly increased secretion. In our analyses, we found several exceptions for extensions at the carboxy-terminus that do not induce elevated E^rns^ secretion. One possibility close to the natural situation is the presence of the E1 membrane anchor region. As described above, the E^rns^-E1 precursor is bound to the lipid bilayer via the hydrophobic carboxy-terminal region of E1. We recently showed that this part of the protein re-orientates after cleavage at the E1/E2 site to a transmembrane configuration [57]. Fusion of this sequence to different carboxy-terminally truncated E1 fragments (which in fact are constructs with internal deletions of the E1-coding sequence) did not lead to increased secretion, but these mutant proteins still showed a severe processing defect as described above (see also [19]). 

**Figure 8:**
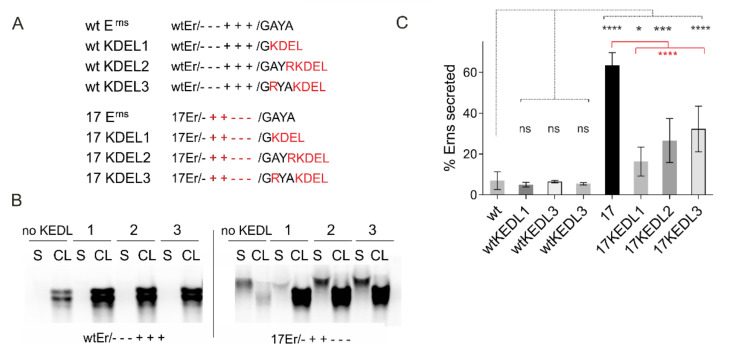
Effect of a KDEL retrieval signal on the secretion of E^rns^ and a mutant thereof. (**A**) Representation of the basic features of the constructs used in the experiments. The upper panel shows the constructs based on the wt sequence of E^rns^ whereas the lower panel lists the constructs based on mutant 17Er (see also legend to Figure 3 and [80] for further information on the mutant). On the left the names of the constructs are given whereas on the right the charge distribution in the amphipathic helix and the carboxy-terminal sequences of the encoded E^rns^ proteins specifying the type of the KDEL modification are shown. Changes with regard to the wt sequence are in red. (**B**) Result of an immunoprecipitation experiment of transiently expressed proteins using E^rns^-specific monoclonal antibody 24/16 and separation of precipitates by SDS-PAGE. Proteins were precipitated either from supernatant (S) or from equivalent amounts of lysate of the transfected cells (CL). On top of the gels the expressed constructs are specified. Left part: constructs based on the wt construct SE^rns^ [19]; right part: constructs based on mutant 17Er. Please note that the secreted proteins exhibit a significantly higher molecular weight due to maturation of carbohydrates in the Golgi apparatus. (**C**) Diagram summarizing the results of three independent experiments quantified via phosphorimager analysis. The names of the constructs are given at the X-axis. The bars represent the amount of secreted protein as percent of total recovered E^rns^. The counts determined for extra- and intracellular E^rns^ were set to 100% expression product as basis for calculation of the secretion value. Error bars are indicated as well as the *p*-Value of E^rns^ mutants compared to E^rns^ wt without KDEL (black lines and asterisks) and of the mutants containing KDEL compared to mutant 17Er without KDEL (red lines and asterisks) with **** representing *p* < 0.0001, * *p* = 0.0109, *** *p* = 0.0002 and ns = not significant.

Another interesting set of constructs were established during analyses aiming at elucidation of the way E^rns^ proteins take within the cell when they are secreted. To get some information on this, we established plasmids coding for E^rns^ with a carboxy-terminal KEDL sequence. KDEL is a retrieval signal, responsible for back-transport from the Golgi apparatus of soluble ER proteins via COP-I vesicles [115,116]. With this approach we wanted to see whether KDEL can prevent secretion of E^rns^. The constructs contained either the wt E^rns^ sequence or mutants thereof. The KDEL-coding sequence was fused to the carboxy-terminus in three different ways, either replacing the last E^rns^ residues (Series 1 with the carboxy-terminal-GAYA motif of E^rns^ replaced by -KDEL) so that also the von Heijne SP cleavage site was eliminated, or fused to the last residue with either a change of -GAYA to -GRYA or -GAYR leading to -GRYAKDEL (Series 2) or -GAYRKDEL (Series 3). The mutations in the latter two series were introduced to prevent removal of the KDEL by SP cleavage. Interestingly, the introduction of KDEL did not have any measurable effect on secretion of wt E^rns^, showing that the extension did not provoke secretion, which was surprising since the last four residues of E^rns^ play an important role in intracellular retention of the protein (see Section 3.1 and Figure 1). Moreover, addition of KDEL to strongly secreted charge exchange mutants resulted in considerably reduced secretion rates, which, however, were still significantly higher than those determined for wt E^rns^ (Figure 8). These findings support the conclusion that E^rns^ is transported on the regular export pathway from ER via the Golgi apparatus to the plasma membrane so that KDEL can orchestrate back-transport from proteins arrived in the Golgi to the ER in considerable amounts.

Another interesting finding in this context was that constructs coding for E^rns^ with three and five extra alanine residues at the carboxy-terminus (SE^rns^-3A and SE^rns^-5A, respectively) showed secretion levels in the same range as wt E^rns^ whereas an extension of 5 E1 amino acids exhibited a secretion level of 40% compared to less than 10% for wt. As a further step we analyzed a construct containing the E^rns^ gene fused to a sequence coding for the 10 amino acids c-Myc tag. Expression of this construct resulted in only significantly less than 10% of the E^rns^-Myc protein in the supernatant whereas E^rns^ with 10 amino-terminal residues of E1 (SE^rns^-E1:10) showed 40% secretion [19]. According to our data this finding cannot be explained by more efficient processing of the c-Myc fusion protein so that the E^rns^ c-Myc fusion protein is most likely less efficiently secreted because this tag leads to less impaired membrane binding and/or interaction with retention relevant partners. Taken together, these results show that a carboxy-terminal extension fused to E^rns^ does not generally lead to a high secretion level. However, processing at the E^rns^ carboxy-terminus is strongly hampered in the absence of a full length E1 showing again the importance of the downstream sequence for cleavage at this site. 

#### 3.6.3. Not All E^rns^ Synthesized in Cells Is Secreted Over Time

**Figure 9:**
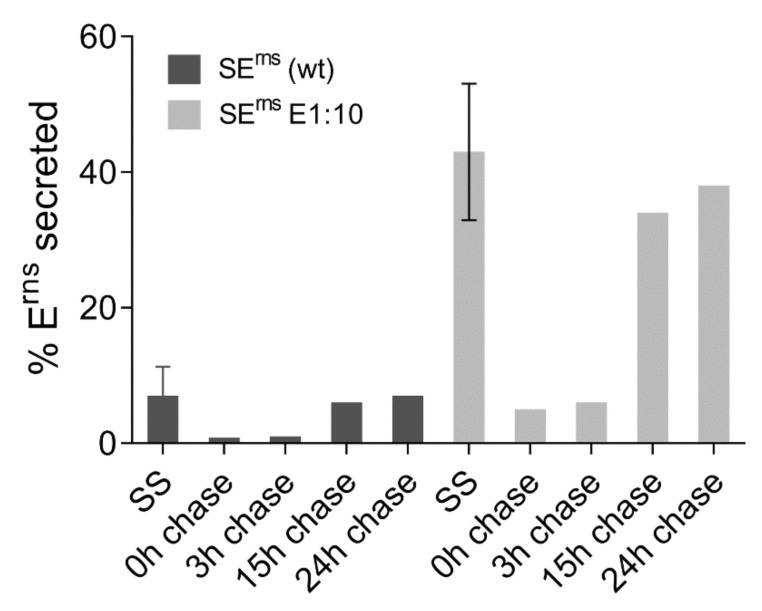
Pulse/chase analysis of E^rns^ secretion. The diagram summarizes the results of pulse chase studies comparing the secretion of wt E^rns^ (SE^rns^) and the super-secretion mutant SE^rns^E1:10 over time. The bars represent the amount of secreted protein as percent of total recovered E^rns^. The counts determined for extra- and intracellular E^rns^ quantified via phosphorimager analysis were set to 100% expression product as basis for calculation of the secretion value. At the X-axis the information on the samples is given with SS standing for steady state (26 h labelling) and for the other samples the time of chase following a 2 h pulse labelling time.

All our published results on E^rns^ secretion were based on steady state analyses reflecting the situation after ca. 24 h. Since all proteins synthesized during this time were labelled it is impossible to distinguish whether proteins synthesized during the first hours are all found in the supernatant or whether a certain percentage of the proteins are bound to secretion whereas the rest stays within the cell. The latter would lead to a stable equilibrium between secreted and retained proteins. To get information on this point we conducted pulse/chase experiments with cells expressing wt E^rns^ or the super secretion mutant SE^rns^-E1:10 [19]. Transfected cells were pulse labelled for 2 h, followed by chase times of 0, 3, 15 and 24 h. As a control, full time labelled samples were prepared. As shown in Figure 9, secreted proteins were difficult to detect for the wt, especially at the early time points. Phosphorimager analysis revealed an increase from less than 1% of secreted product at 0 h chase to 7% at 24 h chase, which is in the same range as the mean steady state value of 6% determined in many experiments. As expected, expression of SE^rns^-E1:10 resulted in significantly higher secretion rates. The quantification resulted in detection of 5% secretion at 0 h chase and a continuous increase up to 38% at 24 h chase, which is again similar to the result obtained with full time labelling (42%). We were not able to further prolong the chase times since the cells started to die around 24 h. The determined secretion rates show that there is an obvious delay in the beginning, which reflects the time the protein needs for passing the secretory pathway. However, the equivalent values determined for full time labelling versus 2 h labelling plus 24 h chase and the very low increase between 15 h and 24 h chase clearly indicate, that the timely change of the secretion level represents a saturation curve. Thus, it can be concluded that E^rns^ secretion is most likely not a constant “bleeding” of protein from the intracellular reservoir but the result of a specific “yes” or “no” fate. Part of the molecules acquire a “yes” configuration while the other end up in a “no” state. These results fit with the finding that the majority of E^rns^ does not cycle freely in the cell but is retained statically (see E^rns^ retention in 3.3). We have no idea at the moment of the molecular switch determining this decision but one can imagine that this equilibrium guarantees the availability of sufficient intracellular E^rns^ for particle formation as well as the necessary level of the virulence factor E^rns^ in the cell free section of an infected animal. 

#### 3.6.4. E^rns^ Is Not Secreted as a Soluble Protein but as Part of Vesicles or Other Larger Structures

The experiment with the KDEL motif fused to the E^rns^ carboxy-terminus revealed that E^rns^ secretion is reduced when its transport from the Golgi apparatus back to the ER is artificially induced. Moreover, the glycosylated E^rns^ protein found in the supernatant has a higher molecular weight than the intracellular protein whereas both proteins comigrate in a gel after PNGase F treatment [18]. This finding can be explained by carbohydrate maturation in the Golgi, which leads to higher molecular weight. Thus, there is significant evidence that secreted E^rns^ stems from protein translocated into the ER and passing the Golgi on its way out of the cell. This is true for both wt and all mutants with increased secretion. One easy model for the mechanism leading to release of E^rns^ into the cell free supernatant would be that the protein for some reason loses membrane binding, which would most likely lead to a structural change of the amphipathic helix resulting in shielding of hydrophobic amino acids. Such a new fold would hinder renewed establishment of membrane binding and thus favor transport of the protein in vesicles along the secretory pathway and finally release it into the cell free medium. A somewhat similar alternative would be that (mis)folding of the amphipathic helix during E^rns^ biosynthesis and processing could prevent establishment of a stable membrane/anchor contact, though this idea would contradict the proposed coordinated process of E^rns^ carboxy-terminus folding, membrane binding and processing. Both of these ideas represent kind of rather low frequent accidents and not a systematic mechanism for generation of a specific equilibrium between retention and secretion. 

**Figure 10:**
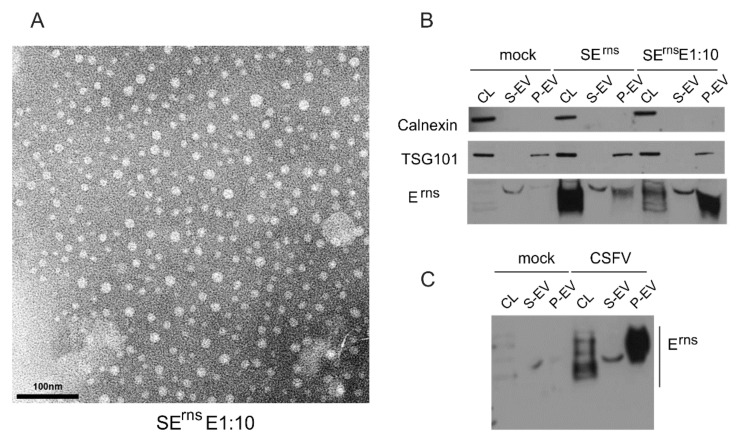
E^rns^ copurifies with extracellular vesicles. (**A**) Electron microscopic picture of a typical preparation of extracellular vesicles from the supernatant of cells expressing SE^rns^E1:10. Differences from this picture with regard to number or morphology were not observed when preparations from cells expressing wt E^rns^ or naive cells or pestivirus infected cells were analyzed. (**B**) Western Blot with samples produced during EV enrichment. Cultures of non-transfected BHK-21 cells infected with Vaccina virus MVA-T7 (mock), and cells expressing E^rns^ wt (SE^rns^) or the super-secretion variant mutant SE^rns^E1:10 served as starting material. Supernatant of the cultures was subjected to vesicle enrichment via the ExoQuick-TC kit resulting in a vesicle containing pellet fraction (P-EV) and a vesicle-free supernatant (S-EV). For control purposes, lysates of the corresponding cells were analyzed (CL). Antibodies against calnexin (upper panel, marker for cell resident proteins), TSG101 (marker for EVs/exosomes, middle panel) and E^rns^ (lower panel) were used for Western blot. (**C**) same as in B but with SK6 cells infected with CSFV Alfort/Tübingen or SK6 mock control.

Another attractive idea not relying on (accidental) loss or prevention of membrane binding is the secretion of E^rns^ as a membrane bound protein. Eukaryotic cells secrete high numbers of different lipid bilayer-surrounded structures [117]. Such vesicles could be loaded with E^rns^ when cells express this protein. Moreover, E^rns^ might be able to induce formation of such vesicles in kind of an irregular budding process since insertion of its long amphipathic helix is prone to induce membrane curvature so that accumulation of a certain number of E^rns^ molecules in one area of a membrane could induce vesicle budding [75,118]. We, therefore, enriched extracellular vesicles (EVs) with the ExoQuick-TC kit designed for isolation of exosomes, a special type of EVs. The supernatant of mock cells as well as cells transiently expressing E^rns^ wt or the strongly secreted fusion protein SE^rns^-E1:10 with 10 residues of E1, was depleted from gross debris by low speed centrifugation and then treated as recommended by the supplier of the kit resulting in a pellet that should contain the purified vesicles (P-EV) and a supernatant depleted of such vesicles (S-EV). Figure 10A shows the results of an electron microscopic analysis of the P-EV pellet of cultures with cells expressing SE^rns^E1:10. All three samples resulted in detection of similar amounts of vesicles with a size of ca. 10−20 nm and equivalent morphology (data not shown). Thus, expression of E^rns^ or its super-secretion variant did not lead to a general increase of EV production or to release of a different type of vesicles. A sample of the lysate of the cells, and aliquots of S-EV and P-EV were analyzed by SDS-PAGE and Western blot (Figure 10B). To control the fractionation success, antibodies against the ER protein calnexin (only present in intracellular membrane compartments) and TSG101 (a marker protein for exosomes) were used. Calnexin was only found in the cell lysate showing that the supernatant did not contain significant amounts of cellular material. TSG101 was detected in cell lysate and the P-EV pellet but not in S-EV, proving that the purification method worked very successfully (Figure 10B). As expected, E^rns^ was absent from the mock control but detected in the lysates of the transfected cells. Importantly, E^rns^ was also clearly detectable in the P-EV but not in S-EV, in which only one band was detected with the E^rns^ antibody that was also present in the mock control. As shown before in other analyses [18,19] wt E^rns^ was mainly found in cell lysate whereas the SE^rns^-E1:10 band was significantly less intense in cell lysate because of heavy secretion (Figure 10B). The secreted protein was detected as a strong band in the P-EV. These results show that E^rns^ is secreted as part of a structure behaving like EVs in the purification procedure. Moreover, the increased secretion of the E^rns^ mutant with extra E1 residues is obviously not an artificial process but follows the same scheme as wt E^rns^. 

**Figure 11:**
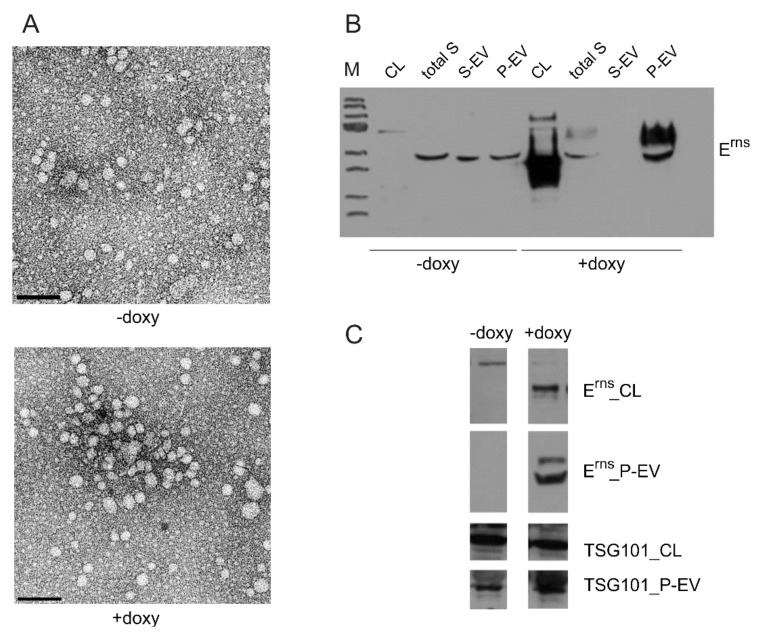
SK6TO_E^rns^ cells stably expressing E^rns^ under control of an inducible promoter secrete E^rns^ in a vesicle bound form. (**A**) Electron microscopic pictures of vesicles enriched from the supernatant of SK6TO_E^rns^ cells with or without doxycycline induction (lower or upper panel, respectively). Bar represents 100 nm. (**B**) Western Blot with samples produced during EV enrichment. Cultures of SK6TO_E^rns^ cells minus or plus doxycycline induction served as starting material. Supernatant of the cultures was either loaded without fractionation (total S) or subjected to vesicle enrichment via the ExoQuick-TC kit resulting in vesicle containing pellet fraction (P-EV) and vesicle-free supernatant (S-EV). For control purposes lysates of the corresponding cells were analyzed (CL). Antibodies against E^rns^ were used for Western blot with samples from the different fractions separated via SDS-PAGE. (**C**) Similar as in (**B**) but including a TSG101 control showing that treatment with doxycycline for induction of E^rns^ expression has no dramatic effect on EV secretion.

Transient expression of E^rns^ via the Vaccinia virus MVA-T7 system seemed not to be the optimal approach for further analyses. We, therefore, analyzed cells infected with CSFV and obtained equivalent results with E^rns^ found only in the lysate of infected cells and the pellet of the EV preparation (Figure 10C). It has, however, to be mentioned that at least part of the extracellular protein in the pellet results from released virus particles, since we could show that infectious virus was copurified with the pellet fraction. As an alternative source of E^rns^-containing EVs we used SK6TO_E^rns^ cells, which express E^rns^ of CSFV from a sequence stably integrated into the cellular genome under the control of a Tet-on promotor inducible by doxycycline. When triggered with doxycycline lysates of these cells contain significant amounts of E^rns^ whereas the protein is hardly detectable in the supernatant due to the rather low secretion level and the dilution effect in the medium (Figure 11). However, in a Western blot with P-EV prepared from the clarified supernatant E^rns^ was detected as a strong band with increased molecular weight compared to the lysate. Again, detection of TSG101 in the P-EV was used as a control for the specific enrichment of vesicles by the preparation procedure. This result was similar to what we found for infected or transfected cells. The electron microscopic analysis once again showed that the P-EV indeed contained vesicles of the characteristic size. Neither number nor morphology were obviously influenced by the doxycycline treatment.

**Figure 12:**
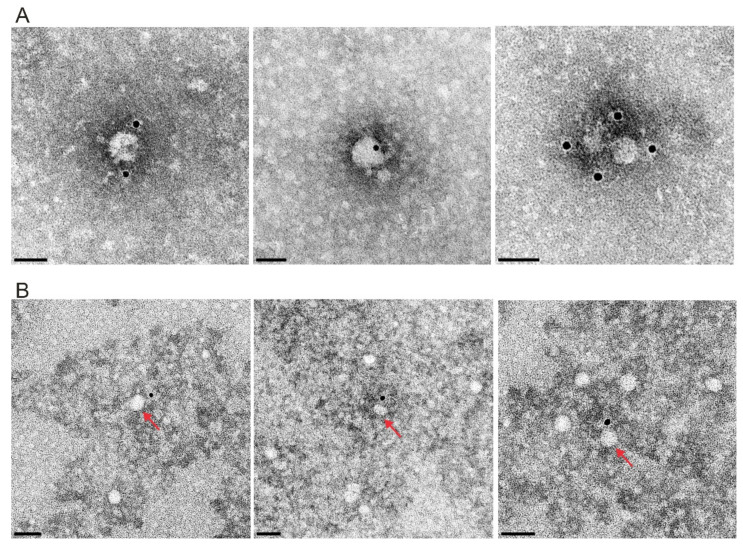
Electron microscopic pictures of immunogold-labelled pestivirus particles and enriched vesicles. CSFV particles enriched from the supernatant of infected SK6 cells by centrifugation (**A**) or vesicles enriched from supernatant of SK6TO_E^rns^ induced with doxycycline were analyzed by immunogold-labelling using E^rns^ specific mab 24/16 and gold-labelled secondary antibody. In (**B**) vesicles with gold grains are highlighted with red arrows. Bars represent 50 nm.

P-EVs prepared from these cells were used to test whether E^rns^ can be detected on the surface of the purified vesicles. To establish the detection method, we started with viruses pelleted from the supernatant of infected cells and stained with the E^rns^ specific monoclonal antibody 24/16 and a gold-labelled secondary antibody (Figure 12). After negative stain gold grains were found close to particles of ca. 40 nm diameter, which fits with the known size of pestivirus virions. Using the same staining method for vesicles isolated from the supernatant of doxycycline triggered SK6TO_E^rns^ with the ExoQuick-TC kit resulted in detection of gold grains close to the roughly 20–30 nm vesicles (Figure 12). Only very few vesicles were labelled which is in part due to the rather inefficient labelling procedure (also virus particles were quite poorly labelled). However, this observation most likely also indicates that only a rather small portion of the vesicles contain (accessible) E^rns^ on their surface. 

### 3.7. Effects of E^rns^ Membrane Anchor Mutations on the Recovery and Viability of Infectious Viruses

Taken together, our mutagenesis analyses proved that a variety of mutations influence the processing at the E^rns^/E1 site as well as the retention/secretion equilibrium of mature E^rns^. With regard to the conserved charged residues in the E^rns^ membrane anchor the influence is quite variable and cannot be foreseen on the basis of the introduced changes showing that these residues are not of crucial importance per se for E^rns^-E1 processing and intracellular retention of E^rns^. Loss of single charged amino acids had only minor or moderate effects, and even mutation of several charged residues did not block E^rns^-E1 processing completely. The maximal effect on E^rns^ secretion rate in consequence of multiple exchanges was in the same range as observed for insertions impairing the amphipathic character of the E^rns^ carboxy-terminus [14] or carboxy-terminal deletions of the E1 moiety in the E^rns^-E1 precursor [19]. The general level of the observed effects on processing and retention, and especially its dependence on the position of the alteration in the amphipathic helix do not support the idea of general importance of the conserved charge distribution. Thus, the reason for their conservation cannot be deduced from the analysis of processing and secretion. We, therefore, tested a selected set of our mutations with regard to their effect on recovery and propagation of CSFV Alfort/Tübingen via our reverse genetics system [32]. We selected mutants with moderate effect on secretion/low change of processing (e.g., mutant 18+++/--- with exchanges D7R/D4R/E1R/R4E/R9E/R6E, mutant 12++-/+-+ with exchanges D7R/D4R/R9E and construct 31--+/+++ with E1R), moderate influence on both secretion and processing (e.g., mutant 32---/-++ = R4E) and strong alteration of both processes (e.g., mutant 27---/--+ with R4E/R9E) (see legend to Figure 3 for nomenclature of the mutants). 

**Figure 13:**
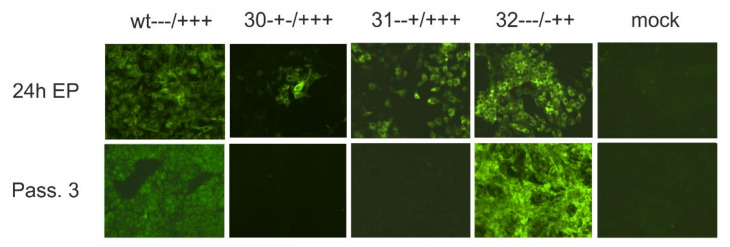
Exchange of charged residues in the E^rns^ membrane anchor can block production of infectious virus. Immunofluorescence analyses conducted after transfection of CSFV genome-like wt RNA or RNA with the given selected exchanges of one triplet coding for a charged amino acid in the carboxy-terminal region of E^rns^. The upper row shows results of analyses conducted ca. 24 h post electroporation and proves that all tested RNAs represent functional replicons. The lower row shows the results obtained after three passages of cell culture supernatant used for infection of fresh cells. Immunofluorescence was done with mab a18 directed against the E2 protein [33]. “-“ represents a negative, “+” a positive charge. Magnification 100×.

For testing less invasive changes, we used single alanine mutations that had mostly shown no significant influence on E^rns^-E1 processing (Table 1). For all mutants, the in vitro transcribed RNAs yielded positive immunofluorescence signals (Figure 13) and thus proved to function as autonomous replicons since input RNA does not lead to detectable protein levels in our system [32]. Freeze/thaw extracts of the transfected cells were used for infection of fresh cells followed by two passages and then analyzed by indirect immunofluorescence for expression of viral proteins. Positive results were obtained for the RNAs with the wt sequence and the mutants with only single exchanges except for mutants 30-+-/+++ (D4R) and 31--+/+++ (E1R). Among mutants with more than one replaced amino acid only the double mutant 27---/--+ (R4E/R9E) allowed recovery of infectious viruses whereas the other mutants and the mock control were negative (Table 1, Figure 13 and [80]).

An interesting question is of course whether the mutations are stable or whether (pesudo)reversion occurs. RNA isolated from cells of the third passage infection with the recovered viruses followed by RT-PCR and nucleotide sequencing revealed changes for mutants 27---/--+, 32---/-++ (R4E), 34---/++- (R6E) and the Ala mutants 35--a/+++, 36---/a++ and 51-a-/+++ (with E1a, R4a and D4a, respectively) (Table 1). These changes were either reversions or pseudoreversions, in which the original charge was present at the mutated position but not the original amino acid. Taken together, these results proved the importance of the charged residues for recovery of infectious viruses with even single charge exchanges interfering with production of infectious pestiviruses [80]. The pseudoreversions underline the role of a certain charge at the respective positions whereas the original amino acid seems not to be crucial. Since some of the tested mutations were stable we tested the replication characteristics of these mutants. Mutants 29+--/+++ (D7R), 33---/-+- (R9E) and 53---/--a (R6a) showed wt growth characteristics, which result further supports that these alterations do not hamper the virus at all [80].

Conserved amino acids are also found at the amino-terminus of E1 [19]. E^rns^ carrying several of these residues at its carboxy-terminus was strongly secreted in contrast to a version with an extension made up of the 10 residue c-Myc tag. We were interested to see how changes affecting these conserved E1 residues go along with recovery and growth of infectious viruses. We, therefore, introduced several mutations into the 5′ terminal part of the E1 coding sequence of SE^rns^-E1. In the first series of three mutants, we changed the residues at Positions 3, 4 and 5 of E1 (P, Y and C) to A, T and S, respectively. A second set contained AAA for PYC (Positions 3 to 5) or EQKL instead of LSP (Positions 1 to 3 plus one extra amino acid) with the EQKL representing the amino-terminal sequence of the c-Myc tag (Table 1). Since after transient expression these exchanges did not lead to significantly impaired E^rns^-E1 processing or E^rns^ secretion rates, conservation of the sequence was not due to a function in processing or E^rns^ membrane binding (not shown). When single site exchanges were tested in our infectious full length cDNA clone transfection of RNA resulted in all cases in recovery of infectious viruses (Table 1). However, sequence analysis revealed reversion of the Y498T and C499S mutants regenerating the Tyr and Cys residues at their original positions. In contrast, the P497A mutation was still present. The triple mutant PYC to AAA was negative when passage to fresh cells was tried, but for the variant with the four c-Myc deduced residues 100% of the cells were infected. Sequence analysis revealed that the recovered virus had regenerated the pro reside at Position 497 (Table 1). Taken together, the amino-terminal residues of E1 are not important for E^rns^-E1 processing when full length E1 is present, but the conserved residues play a role in production of infectious viruses.

### 3.8. Mutations of Certain Charged Amino Acids in the E^rns^ Membrane Anchor Impair Virus Particle Formation or Release

**Figure 14:**
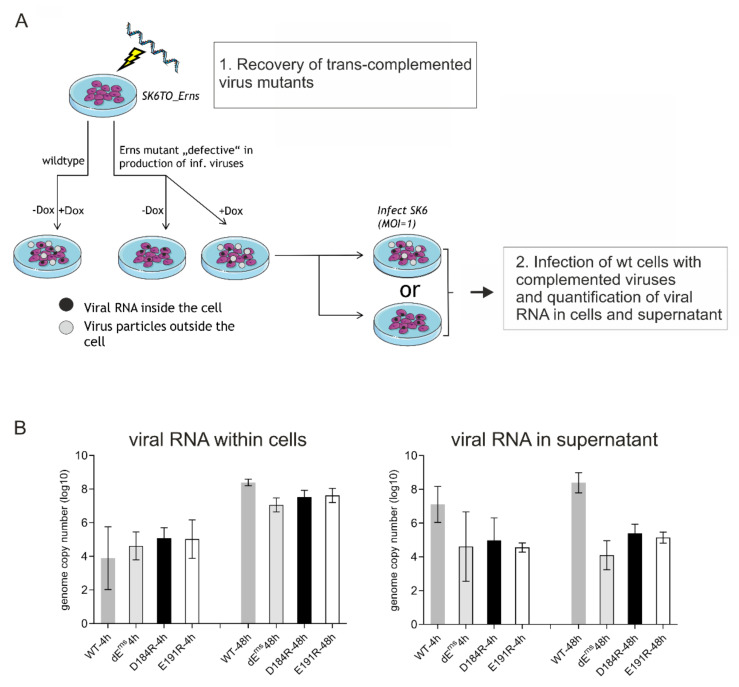
Determination of viral RNA levels in cells and cell culture supernatant. (**A**) Schematic presentation of the method used for propagation of mutant viruses. Step 1: SK6_TO cells were transfected via electroporation and E^rns^ production (by the cells) was induced with doxycycline. Viral particles were recovered from wt or defective mutant RNAs due to the trans complementation with wt E^rns^. Step 2: The recovered infectious viruses were used in single cycle experiments for infection. Viral RNA was harvested from cells and supernatant and then used for quantification of viral RNA displaying the wt sequence or an E^rns^ deletion mutant or mutants with deleterious exchanges. Viral RNA in the supernatant should result from secretion of viral particles shown here as gray dots. (**B**) Results of viral RNA quantification via qRT-PCR in lysates (left) or equivalent amounts of supernatant of SK6 cells infected with wt CSFV or stocks of mutants propagated in complementing SK6TO_E^rns^ cells. Infection was done with an MOI of one. Harvest for total RNA isolation was done separately for cells and supernatant at 4h or 48 h p.i. and the viral genome copy number was determined via real time RT-PCR. The bar diagrams summarize the results of three independent experiments showing the mean log10 values of the viral genome copy numbers with standard deviation indicated.

There are different possible explanations for the defect resulting from changes affecting the amphipathic helix at the E^rns^ carboxy-terminus. These explanations are connected with different putative functions of E^rns^ or of its carboxy-terminus for the viral life cycle. It is known that E^rns^ binds to carbohydrate structures on the surface of target cells and thus initiates the attachment process [119,120,121,122]. One could, therefore, speculate that the integrity of the E^rns^ carboxy-terminus is crucial for the infection process. Alternatively, the amphipathic helix plays an essential role in the pestivirus assembly, budding or release process. The obvious difference between the two alternatives is the presence of packaged viral RNA outside of the cells in the former case whereas the second possibility would lead to retention of viral RNA within the cells. To answer the question whether our dead non-infectious mutants were able to package and secrete viral RNA we developed an assay system allowing to introduce significant amounts of genomic wt or mutant RNA into cells and monitor the levels of intra- and extracellular viral RNA over time (Figure 14). To this end, we transfected SK6TO_E^rns^ cells with the different in vitro transcribed genome-like RNAs. Stimulation of these cells with doxycycline provides functional wt E^rns^ in trans for virion production and secretion. In addition to the wt RNA and the mutants D184R and E191R (Mutants 30 and 31, respectively; nomenclature of mutants described in the legend to Figure 3) we included in these assays a deletion mutant lacking the complete E^rns^ sequence (except for the signal sequence necessary for translocation into the ER). Immunofluorescence analyses proved that significant amounts of infectious viruses had been generated regardless which RNA had been transfected. After incubation of the transfected cells for 48 h, a virus containing extract was prepared through freeze/thaw cycles. This extract was cleared and used for infection of wt SK6 cells. The immunofluorescence analysis of those wt SK6 cells proved the recovery of infectious viruses for all extracts derived from transfected SK6TO_E^rns^ cells. However, only the wt virus without any mutation could spread in the cell culture since the mutants were not provided with functional E^rns^ when propagated in wt SK6 cells. In the further course of the experiment extracts of the infected wt SK6 cells were prepared and used to infect fresh cells. As expected, the IF analysis was negative except for the wt control stemming from the cells originally transfected with RNA of wt CSFV Alfort-Tübingen. 

Thus, this method allows the generation of complemented viruses suitable for one cycle infection experiments. The recovered viruses from the SK6TO_Erns cells were titrated and served as virus stocks for the further experiments

When SK6_TO cells were transfected with the RNA, viral protein was clearly detected via immunofluorescence analysis. However, when no doxycycline was added infection of fresh cells with freeze/thaw extracts prepared from these first cycle infected cells resulted in negative immunofluorescence results for the mutants. Only the wt sample was positive. The absence of infectious virus mutants had to be expected from the earlier results with lysates of electroporated wt SK6 cells [80]. When doxycycline was added to the electroporated complementing cells infectious viruses were recovered also for the mutants. Infection of wt SK6 cells with freeze/thaw extracts of the electroporated cells resulted in detection of viral RNA within the cells and the amount of this RNA increased significantly from 4 to 48 h (Figure 14B). The stronger increase of viral RNA observed for the wt sample presumably results from the ability of this RNA to produce infectious progeny virus that can infect cells that were not infected in the first cycle with only an MOI of one. In any case, the autonomous replication of all four RNAs within the cells was clearly demonstrated by this result. When regarding the supernatant, the amount of wt RNA outside of the cells was surprisingly higher compared to the different mutants although equivalent TCID_50_ were used for infection. We cannot explain this result, since 4 h p.i. seem to be very early for export of viral RNA but this result was constantly obtained in different independent experiments. The amount of wt RNA in the supernatant increased considerably from 4 to 48 h. In contrast, the RNA levels of the mutants stayed in the same range over this time. This observation strongly supports the conclusion that these viral RNAs cannot support the production and/or release of particles containing viral RNA. 

## 4. Discussion

The E^rns^ protein of pestiviruses represents a very special product of viral evolution, a kind of a swiss army knife that integrates a role as structural protein essential for generation of infectious viral particles and a virulence factor function important for controlling the host’s innate immune response to pestivirus infection. With regard to both of these functions, our knowledge of the detailed molecular mechanisms is very poor, despite considerable and successful efforts taken to characterize this protein even on the structural level [5,6,9,11,12,16,20,21,23,123,124,125,126]. Deletion of the E^rns^ coding sequence from the viral genome prevents the generation of infectious viruses (this report and [6,95,96,119]). This could argue in favor of an E^rns^ dependent step during infection. This idea was supported by the observations that E^rns^ binds to carbohydrate structures on the cell surface and addition of E^rns^ to culture medium inhibits infection [119,120,121,122,127]. Accordingly, the idea was put forward that E^rns^ represents an attachment protein establishing the first contact to target cells. However, in pseudo-typed retroviruses or VSV E1 and E2 were sufficient for infection indicating that E^rns^ might also be dispensable for the basic infection process of pestiviruses [3,4]. In this case, the blocking of virus entry by excess soluble E^rns^ might be due to other processes like, e.g., steric hinderance. Work on the carboxy-terminal membrane anchor of E^rns^ shed some light on putative functions of E^rns^. The respective experiments were initiated because part of the E^rns^ protein synthesized within cells is secreted into the supernatant [13], a fact that fits well to the results of sequence analyses indicating that E^rns^ is bound to lipid bilayers via an unusual structure. Detailed mutational and biophysical analyses revealed that E^rns^ is bound to membranes via a long amphipathic helix located at its carboxy-terminus [14,16,17,36].

The unusual membrane anchor is also responsible for retention of the protein in the ER [37]. The static retention of E^rns^ in the ER suggests a specific interaction with the lipid environment instead of the classical retrieval receptor retention that can be found for proteins using the KDEL or KKXX signals. The direct interaction with lipids is proven through the fact that peptides derived from the E^rns^ carboxy-terminus were almost always pulled down with lipid vesicles [17] and that different experiments showed that the E^rns^ carboxy-terminus can also bind cytosolic proteins to the cytosolic side of the membrane [14,17,36]. Nevertheless, different substitutions had diverse impact on the retention, despite negligible effects on membrane association hinting that simple lipid interaction due to the amphipathic character is not the only requirement, but that it might be sensitive for specific lipid compositions or other factors in the ER. These could then also play a role in the decision whether the protein is retained or will be secreted. 

Amphipathic structures are well known to confer (transient) membrane interaction of cytoplasmic proteins. To our knowledge, proteins found on the surface of eukaryotic cells have so far not been described to be anchored by such a structure except for two viral envelope proteins, namely E^rns^ and the GP3 of the arterivirus porcine reproductive and respiratory syndrome virus (PRRSV) [128]. Both of these proteins are secreted from the infected cells. This is hypothesized to be due to the unusual membrane anchor and regarded as functionally important. Disturbance of the amphipathic character of E^rns^ leads not only to reduced E^rns^-E1 processing and E^rns^ secretion but also prevents production of infectious virus particles [14,18]. However, it is difficult to draw conclusions from these results since the influence of these changes on processing and retention of the protein is very strong. Thus, a processing defect could block particle formation or a retention defect could separate mature E^rns^ from the site of budding and thereby impair morphogenesis of the virions. Much milder effects on processing and secretion were observed for mutations affecting conserved charged amino acids in the E^rns^ carboxy-terminus or the highly conserved residues at the amino-terminus of E1 (this report and [80]). Some exchanges affecting these residues influence E^rns^ secretion only moderately and have no significant or a very slight effect on processing [80]. Importantly, some of these charged residue mutants like 33, 50, 52 and 53 (for nomenclature of mutants refer to legend of Figure 3) as well as the P497A exchange in the amino-terminal sequence of E1 allowed recovery of virus mutants that grow equally well as the wt (Table 1 and [80]). Mutation 33 exhibits similar effects on processing and secretion as Mutations 30 and 31 that block the recovery of infectious viruses so that one can conclude that problems with processing and secretion are at least not the main reasons for this block. Accordingly, these mutations were the most interesting for further analyses.

The fact that a mutation preventing recovery of infectious viruses is located in a protein domain shielded at least in part by the lipids of the membrane makes it quite unlikely that the absence of infection is due to inhibition of attachment to the cell surface. Our real time RT-PCR results presented here strongly suggest that the block is associated with prevention of budding and/or release of virus particles. Thus, it can be hypothesized that E^rns^ and especially its carboxy-terminus has a special function in (one of) these processes. Binding of amphipathic helices to membranes imposes pressure on the lipid bilayer that results in curvature [75,117,118]. It could, therefore, well be that the E^rns^ carboxy-terminal amphipathic helix plays a central role in induction of virus budding but this hypothesis has to be analyzed in further experimental work. 

Budding induced by insertion of the E^rns^ amphipathic helix into the ER membrane could also explain the observation that E^rns^ in contrast to mutually accepted hypotheses is not secreted as an isolated molecule but in a membrane bound form. Our analyses indicate that in fact E^rns^-containing vesicles of about half the size of pestivirus virions are secreted from infected cells or cells expressing the protein. Even though the labelling with E^rns^ antibodies was rather poor our results support the conclusion that such vesicles carry E^rns^ on the outside surface. This would fit very well with a budding-like mechanism leading to secretion of E^rns^. The way out of the cell taken by these vesicles is most likely the secretory pathway, since the secreted protein carries EndoH resistant carbohydrates that result from processing during the passage through the Golgi apparatus. Moreover, addition of KDEL to the carboxy-terminus of E^rns^ significantly reduces secretion of the protein, which, according to the underlying mechanism, should be due to transport from the Golgi back to the ER via COP-I vesicles. KDEL is usually responsible to ensure back-transport of soluble ER protein, which at first glance seems to contradict the recycling of vesicle bound proteins but one has to keep in mind the membrane topology of E^rns^ with both termini presented in an “outside” configuration [14]. Thus, the KDEL receptor could interact with the vesicle bound E^rns^.

Eukaryotic cells secrete a variety of vesicles mostly via budding at the plasma membrane or into multivesicular bodies (MVDs) [117]. Purification of specific types of such vesicles is mainly based on physical properties with size being a major criterium. The ExoQuick-TC kit is known to be very specific for enrichment of vesicles exhibiting the features of exosomes so that we can conclude that secreted E^rns^ has to be part of vesicles that are exosomes or copurify with exosomes even though the vesicles we find seem a bit smaller than exosomes (20–30 nm versus 30–100 nm [129,130]). Smaller vesicles of 20–50 nm have also been described to be present in cell supernatant but their origin is still unclear [131,132]. A diameter at least close to the characteristic size of exosomes was proven by electron microscopic analysis of our preparations and the presence of the exosomal marker TSG101 [129] in the exosomal pellet shows that the procedure enriches these vesicles. However, copurification of E^rns^ with vesicles displaying features of exosomes does not necessarily prove that E^rns^ is secreted as an exosomal protein. Exosomes are derived from multivesicular bodies (MVBs) [129,130] and thus do not pass the Golgi apparatus. Accordingly, secretion via MVBs would contradict the above described features of secreted E^rns^. It, therefore, seems more likely that our preparations contain different types of vesicles with similar biophysical properties but secreted via different pathways. It is important to mention that both TSG101 and E^rns^ are only found in the vesicle containing pellet of the preparations but not the supernatant. This proves the efficiency of the purification procedure so that we can conclude that secreted E^rns^ is exclusively found in vesicle-bound form but not as a soluble protein. 

The finding that E^rns^ is secreted as a component of vesicles has to be taken into account when thinking of the mechanisms underlying the second function of E^rns^ as a virulence factor. The E^rns^ RNase activity represents the key feature in this context [5,6]. Destruction of this enzymatic function via mutagenesis results in viable viruses that replicate normally in tissue culture but show a high degree of attenuation in the natural hosts [5,6]. Moreover, the E^rns^ RNase is one component of a complex strategy resulting in establishment of persistent infection as best studied for BVDV [23]. There is good evidence that E^rns^ targets the Type I/III interferon system [23,24,124,125] but a still unanswered question concerns the detailed task of the RNase in this process. In other words, it is not known which RNA is degraded to obtain the observed effects. Our knowledge on synthesis, processing and topology of E^rns^ led to the conclusion that the RNase cannot be active in virus replication within the infected cell since active RNase is formed and stays within intracellular compartments and therefore is absent from the cytoplasm. This makes also sense when taking into account that the RNase is highly active in degrading the viral genomic RNA once these two molecules get into contact [11]. Moreover, pestiviruses are using another factor, N^pro^, for impairing the interferon response elicited within infected cells [133,134,135,136,137,138,139]. Regarding all these points together with the partial secretion of E^rns^, the obvious and logical conclusion is that the E^rns^ secreted into the cell free supernatant represents the virulence factor [13,20,21,24,124]. This widely accepted hypothesis fueled the investigation of the mechanisms leading to E^rns^ secretion. Of course, the unusual membrane anchor of E^rns^ was of key interest in this context [14,16,36]. As mentioned above, the GP3 envelope protein of PRRSV is the only further surface protein anchored via an amphipathic helix [128]. This type of membrane anchoring is believed to be responsible for partial secretion of both of these proteins ensuring an equilibrium between secretion and retention. This equilibrium is necessary to release a significant amount of these proteins into the supernatant and at the same time retain enough of these structural components for virion formation at the site of virus budding within the cell. We have shown that the amphipathic character of the anchor as well as certain features of the primary amino acid sequence are important for this equilibrium. Moreover, we could show here that E^rns^ secretion is not due to constant bleeding of the molecules from the cell. Instead, a so far unknown molecular “yes or no” switch decides whether E^rns^ molecules are secreted or not. Another surprising result showed that E^rns^ is not secreted as an individual soluble molecule but bound to membrane vesicles. The molecular basis for this controlled formation and secretion of E^rns^ containing vesicles is still obscure and difficult to connect with functional aspects. It was, however, published that the secreted product can be taken up by cells and degrade RNA located in endosomes, which could be an interesting molecular mechanism [124]. What is still missing is a clear clue to where the secreted protein executes its activity to help blocking the IFN-1/3 response to virus infection. Python and co-workers [140] showed that plasmacytoid dendritic cells (pDCs) are activated via contact to CSFV-infected cells to secrete Type 1 interferon, apparently via transfer of viral RNA to the pDCs. This activation was blocked by E^rns^ with the inhibition depending on intact RNase activity of E^rns^ in intracellular compartments. Even though it has not been analyzed whether this effect is executed by membrane bound or free E^rns^ it is tempting to speculate that transfer of E^rns^ on vesicles is responsible for repressing the innate response of the pDCs. In summary, even though we have obtained a significant amount of knowledge over the years, there is still a considerable workload ahead to answer these central questions. These investigations should also elucidate the functional role of the conserved residues in the E^rns^ membrane anchor with the positioning of charges able to form a charge zipper or a set of five highly conserved glycine residues that play only a marginal role for membrane binding/retention and processing but seem again to be important for generation of infectious viruses. As stated in the title of this article, there is much more to learn from the E^rns^ carboxy-terminus than a way of membrane anchoring. 

## Figures and Tables

**Table 1:** Summary of the results of experiments conducted with the infectious CSFV cDNA clone. Column 1 lists the number of the individual mutant (same as in the Figures). Column 2: amino acid exchanges encoded by the construct (see legend to Figure 3 for nomenclature of the mutations); Column 3: charge distribution (“a” represents exchange for alanine); Column 4: determined E^rns^-E1 processing efficiency; Column 5; determined E^rns^ secretion level; Column 6: functionality as replicon; Column 7: recovery of infectious virus, yes or no; Column 8: sequencing results after isolation of RNA from third passage cells and RT-PCR amplification of CSFV sequence, numbering code as in Column 2 and Figure 1; Column 9: charge distribution pattern in E^rns^ of the recovered virus. The last five lines present mutants not affecting the E^rns^ membrane anchor but the conserved residues at the amino-terminus of E1. Therefore, charge patterns are not given here. The positions given for these five mutants refer to the position of the amino acids in the polyprotein starting with the Met of N^pro^ as Number 1, and thus is different from the amino acid positions related to the E^rns^ protein given in Figure 3. n.t. = not tested; n.a. = not applicable. Yellow background color: no virus recovered, blue background color: recovered viruses represent revertants or pseudorevertants. The green color in the last line highlights the residue that showed reversion.

Construct	Mutation	Charge	Precursor	Secretion	Replicon	Inf. Virus	Seq	Charge Rec. Virus
wt	-	---/+++	14%	7%	yes	yes	wt	---/+++
ΔE^rns^	deletion of E^rns^ gene	n.a.	n.a.	n.a.	yes	no	n.a.	n.a.
12	D7R/D4R/R9E	++-/+-+	19%	29%	yes	no	n.a.	n.a.
18	D7R/D4R/E1R/R4E/R9E/R6E	+++/---	30%	43%	yes	no	n.a.	n.a.
27	R4E/R9E	---/--+	44%	63%	yes	yes	4K/9R	---/+++
29	D7R	+--/+++	24%	5%	yes	yes	7R	+--/+++
30	D4R	-+-/+++	23%	26%	yes	no	n.a.	n.a.
31	E1R	--+/+++	16%	28%	yes	no	n.a.	n.a.
32	R4E	---/-++	41%	26%	yes	yes	4K or 4R	---/+++
33	R9E	---/+-+	21%	29%	yes	yes	9E	---/+-+
34	R6E	---/++-	22%	28%	yes	yes	6K	---/++-
								
50	D7a	a--/+++	14%	n.t.	yes	yes	7a	a--/+++
51	D4a	-a-/+++	16%	n.t.	yes	yes	4D	---/+++
35	E1a	--a/+++	12%	n.t.	yes	yes	1E	---/+++
36	R4a	---/a++	19%	n.t.	yes	yes	4R	---/+++
52	R9a	---/+a+	16%	n.t.	yes	yes	9a	---/+a+
53	R6a	---/++a	n.t.	n.t.	yes	yes	6a	---/++a
								
E1/3	P497A	n.a.	n.t.	n.t.	yes	yes	497A	n.a.
E1/4	Y498T	n.a.	n.t.	n.t.	yes	yes	498Y	n.a.
E1/5	C499S	n.a.	n.t.	n.t.	yes	yes	499C	n.a.
E1/3.4.5	PYC497-499AAA	n.a.	n.t.	n.t.	yes	no	n.a.	n.a.
E1/1.2.3.+	LSP495-497EQKL	n.a.	n.t.	n.t.	yes	yes	EQKP	n.a.

## Data Availability

All data are included in the manuscript.
